# Surface Expression, Function, and Pharmacology of Disease-Associated Mutations in the Membrane Domain of the Human GluN2B Subunit

**DOI:** 10.3389/fnmol.2018.00110

**Published:** 2018-04-06

**Authors:** Vojtech Vyklicky, Barbora Krausova, Jiri Cerny, Marek Ladislav, Tereza Smejkalova, Bohdan Kysilov, Miloslav Korinek, Sarka Danacikova, Martin Horak, Hana Chodounska, Eva Kudova, Ladislav Vyklicky

**Affiliations:** ^1^Department of Cellular Neurophysiology, Institute of Physiology of the Czech Academy of Sciences (CAS), Prague, Czechia; ^2^Department of Physiology, Faculty of Science, Charles University, Prague, Czechia; ^3^Institute of Organic Chemistry and Biochemistry, Czech Academy of Sciences (CAS), Prague, Czechia

**Keywords:** NMDA receptor, GluN2B, *de novo* missense mutations, neuropsychiatric disorder, neurosteroids

## Abstract

N-methyl-D-aspartate receptors (NMDARs), glutamate-gated ion channels, mediate signaling at the majority of excitatory synapses in the nervous system. Recent sequencing data for neurological and psychiatric patients have indicated numerous mutations in genes encoding for NMDAR subunits. Here, we present surface expression, functional, and pharmacological analysis of 11 *de novo* missense mutations of the human hGluN2B subunit (P553L; V558I; W607C; N615I; V618G; S628F; E657G; G820E; G820A; M824R; L825V) located in the pre-M1, M1, M2, M3, and M4 membrane regions. These variants were identified in patients with intellectual disability, developmental delay, epileptic symptomatology, and autism spectrum disorder. Immunofluorescence microscopy indicated that the ratio of surface-to-total NMDAR expression was reduced for hGluN1/hGluN2B(S628F) receptors and increased for for hGluN1/hGluN2B(G820E) receptors. Electrophysiological recordings revealed that agonist potency was altered in hGluN1/hGluN2B(W607C; N615I; and E657G) receptors and desensitization was increased in hGluN1/hGluN2B(V558I) receptors. The probability of channel opening of hGluN1/hGluN2B (V558I; W607C; V618G; and L825V) receptors was diminished ~10-fold when compared to non-mutated receptors. Finally, the sensitivity of mutant receptors to positive allosteric modulators of the steroid origin showed that glutamate responses induced in hGluN1/hGluN2B(V558I; W607C; V618G; and G820A) receptors were potentiated by 59–96% and 406-685% when recorded in the presence of 20-oxo-pregn-5-en-3β-yl sulfate (PE-S) and androst-5-en-3β-yl hemisuccinate (AND-hSuc), respectively. Surprisingly hGluN1/hGluN2B(L825V) receptors were strongly potentiated, by 197 and 1647%, respectively, by PE-S and AND-hSuc. Synaptic-like responses induced by brief glutamate application were also potentiated and the deactivation decelerated. Further, we have used homology modeling based on the available crystal structures of GluN1/GluN2B NMDA receptor followed by molecular dynamics simulations to try to relate the functional consequences of mutations to structural changes. Overall, these data suggest that *de novo* missense mutations of the hGluN2B subunit located in membrane domains lead to multiple defects that manifest by the NMDAR loss of function that can be rectified by steroids. Our results provide an opportunity for the development of new therapeutic neurosteroid-based ligands to treat diseases associated with hypofunction of the glutamatergic system.

## Introduction

The N-methyl-D-aspartate receptor (NMDAR) is a heteromeric ligand-gated ion channel permeable to Ca^2+^ that is expressed in neurons and glia. Virtually all brain and spinal cord circuits rely on excitation evoked by transient activation of NMDARs by glutamate, giving rise to NMDAR excitatory postsynaptic currents (EPSCs) to regulate physiological functions. In addition, NMDAR signaling is implicated in various forms of synaptic plasticity (Dingledine et al., 1999; Lynch, 2004; Traynelis et al., 2010; Huganir and Nicoll, 2013).

NMDAR is composed of two obligatory subunits (GluN1) and two variable ones, which can include either GluN2(A-D) or GluN3(A-B) (Traynelis et al., 2010). The GluN1 subunit is expressed throughout the central nervous system, whereas the four subtypes of GluN2(A-D) subunits have differential temporal and spatial expression patterns (Akazawa et al., 1994; Monyer et al., 1994). Expression of the GluN2B subunit (encoded by *GRIN2B* gene) dominates during rapid cortical synaptogenesis in late embryogenesis and early postnatal development, and its expression level starts to decline after birth in most brain regions (Hall et al., 2007). Several lines of evidence indicate that GluN2B subunit plays an important role in brain development, circuit formation, differentiation, and synaptic plasticity (Cohen and Greenberg, 2008). The importance of this subunit is stressed by the neonatal lethality of *GRIN2B* knock-out mice (Kutsuwada et al., 1996).

NMDAR dysfunction is implicated in a variety of nervous system disorders. Early studies indicated that glutamate triggers excitotoxicity (Olney, 1969). This form of neuronal death is dependent on calcium influx through NMDARs (Choi, 1987) and is implicated in neurological disorders such as neurodegenerative diseases, stroke, epilepsy, and traumatic brain injury (Parsons and Raymond, 2014).

Recent advances in high-throughput DNA sequencing have allowed examination of the prevalence of mutations in genes encoding NMDAR subunits among afflicted individuals. While the gene encoding for the GluN2B subunit is the most frequently *de novo* mutated *GRIN* gene in psychiatric and neurodevelopmental disorders, *de novo* mutations of *GRIN1* and *GRIN2A* genes encoding for GluN1 and GluN2A are less frequent and, interestingly, in *GRIN2C, GRIN3A*, and *GRIN3B*, only rare truncating mutations affecting both healthy individuals and patients suffering from neurodevelopmental disorders have been reported (Tarabeux et al., 2011; Soto et al., 2014).

*GRIN2B* variants—*de novo* nonsynonymous mutations, missense, nonsense, frame shift, or splice site mutations—were identified in individuals from patient cohorts with defined neurodevelopmental and psychiatric disorders such as intellectual disability, developmental delay, autism spectrum disorder, epileptic encephalopathy, schizophrenia, and to a lesser extent, attention deficit hyperactivity disorder, cerebral visual impairment, and Alzheimer's disease, as has been recently reviewed (Soto et al., 2014; Burnashev and Szepetowski, 2015; Hu et al., 2016). *GRIN2B* variants were found in the amino-terminal domain (ATD), ligand-binding domain (LBD), transmembrane domain (TMD), and carboxyl-terminal domain (CTD); rare *de novo* mutations in the ATD and CTD, but not the LBD and TMD domains, are present in the exomes of a large control population sample (Lek et al., 2016). This implies that mutations in the LBD and TMD domains may significantly alter the NMDAR channel properties. Indeed, in line with this hypothesis are results of functional examination of disease-causing mutations in the LBD (Adams et al., 2014; Lemke et al., 2014; Swanger et al., 2016). In the case of the TMD, the connections between genetic variation and consequences for the NMDAR channel function and/or trafficking are much less understood.

In this study, our goal was to elucidate the consequences of NMDAR mutations found in individuals diagnosed with neurodevelopmental disorders, in regard to the receptor-channel function and surface expression. We investigated 11 disease-associated variants in the *GRIN2B* gene located in the TMD region of the GluN2B subunit which are found as heterozygous *de novo* missense mutations. We found that some diheteromeric variants of the NMDAR have markedly decreased probability of channel opening and altered receptor surface expression. In addition, the effects of two structurally different neurosteroids, 20-oxo-pregn-5-en-3β-yl sulfate (PE-S; pregnenolone sulfate) and androst-5-en-3β-yl hemisuccinate (AND-hSuc), were tested as a pharmacological tool to correct the impaired function of mutated NMDARs.

## Methods

### Transfection and maintenance of cells

Human embryonic kidney (HEK293T) cells (American Type Culture Collection, ATTC No. CRL1573, Rockville, MD, USA) were cultured in Opti-MEM I (Invitrogen, Carlsbad, CA, USA) with 5% fetal bovine serum (FBS; PAN Biotech, Aidenbach, Germany) at 37°C in 5% CO_2_. One day before transfection, cells were plated in 24-well plates at a density of 2 × 10^5^ cells per well. The next day, the cells were transfected with expression vectors containing human wild-type (WT) NMDAR subunits hGluN1-1a (GenBank accession no. NP_015566) and hGluN2B (GenBank accession no. NP_000825) (both genes were generous gifts from Prof. S. Traynelis, Emory University School of Medicine, Atlanta, GA) (Hedegaard et al., 2012) and green fluorescent protein (GFP; pQBI 25, Takara, Tokyo, Japan) genes. Briefly, equal amounts (0.2 μg) of cDNAs encoding for hGluN1, hGluN2B and GFP were mixed with 0.6 μL of MATra-A Reagent (IBA, Göttingen, Germany) and added to confluent HEK293T cells cultured in 24-well plates. After trypsinization, the cells were resuspended in Opti-MEM I containing 1% FBS supplemented with 20 mM MgCl_2_, 3 mM kynurenic acid, 1 mM D,L-2-amino-5-phosphonovaleric acid and 1 μM ketamine, and plated on 30 mm collagen and poly-L-lysine-coated glass coverslips. Transfected cells were revealed by GFP epifluorescence.

Site-directed mutagenesis of the gene encoding for hGluN2B subunit was performed using the QuikChange II XL Site-Directed Mutagenesis Kit (Agilent Technologies, Santa Clara, CA, USA) in accordance with the instructions of the manufacturer using manually designed primers purchased from Sigma-Aldrich. DpnI-treated product of PCR reaction was transformed into ultracompetent XL10-Gold *E.coli* cells, positive clones were selected, and DNA plasmids were isolated using High-Speed Plasmid Mini Kit (Geneaid, New Taipei City, Taiwan) according to the manufacturer instructions. All mutations were verified by DNA sequencing (GenSeq, Benesov u Prahy, Czech Republic and/or Eurofins Genomics, Germany). Amino acids are numbered according to the full-length protein, including the signal peptide, with the initiating methionine as number 1.

### Transport

#### Heterologous cell culture

African Green Monkey kidney fibroblast (COS-7) cells were grown in Minimum Essential Medium with Earle's salts (Thermo Fischer Scientific) with 10% FBS (v/v), as described previously (Kaniakova et al., 2012).

#### Expression vectors

The human version of the cDNAs encoding for the full-length GluN1-1a subunit tagged with yellow fluorescent protein (YFP-hGluN1) was generated by the QuikChange II XL site-directed mutagenesis kit according to the manufacturer's instructions, and the construct was verified by DNA sequencing.

#### Immunofluorescence microscopy

COS-7 or HEK293T cells were transfected in 12-well plates with a total of 1.8 μg of cDNAs (in case of co-transfection, equal amounts of cDNAs containing YFP-hGluN1 and hGluN2B subunits were used) and 4 μl Lipofectamine™ 2000 (Thermo Fischer Scientific), as described previously (Kaniakova et al., 2012). COS-7 cells were preferred for microscopy because they attach well to glass coverslips while extensive washing procedures are being performed. HEK293T cells that attach to glass less firmly were used to confirm differences in the surface expression observed on COS-7 cells. After 24–36 h, the cells were washed in PBS, and then incubated on ice for 10 min in blocking solution containing PBS and 10% normal goat serum (v/v). After this blocking step, the cells were labeled with primary rabbit anti-GFP (Merck Millipore; Burlington, MA, USA; 1:1,000) and secondary goat anti-rabbit Alexa Fluor® 555 (Thermo Fischer Scientific; 1:1,000) antibodies. Antibodies were diluted in the blocking solution and the cells were incubated with each antibody for 30 min. The cells were then washed twice in PBS, fixed in 4% paraformaldehyde in PBS (w/v) for 20 min, and mounted using ProLong Antifade reagent (Thermo Fischer Scientific). Images were taken on an Olympus Cell^∧^R fluorescence microscope, and the intensity of the surface and total fluorescence signals was analyzed using ImageJ software, as described previously (Lichnerova et al., 2014).

#### Quantitative assay of surface and total expression

COS-7 cells were prepared as described in the Immunofluorescence Microscopy section. After 38–40 h, COS-7 cells were washed with PBS, fixed for 15 min in 4% paraformaldehyde (w/v) in PBS and incubated for 1 h in PBS containing 0.2% bovine serum albumin (BSA; w/v) without (surface expression) or with (total expression) 0.1% Triton X-100 (TX-100; v/v). Cells were incubated in a primary rabbit anti-GFP antibody (Millipore, Billerica, MA; 1:500 for surface expression and 1:1,000 for total expression) diluted in PBS with 0.2% BSA for 1 h. After being washed in PBS, cells were incubated with a secondary antibody (horseradish peroxidase-conjugated donkey anti-rabbit IgG; GE Healthcare, UK; 1:1,000) for 1 h and washed in PBS. Next, 400 μl of ortho-phenylenediamine (OPD; final concentration 0.4 mg/ml) dissolved in phosphate-citrate buffer containing sodium phosphate (Sigma, St. Louis, MO) was added to cells for 30 min (surface expression) or 15 min (total expression). The color reaction was terminated with 100 μl of 3 M HCl and the optical density was determined at 492 nm using a Personal Densitometer SI (GE Healthcare, Pittsburgh, PA). The average background signal measured from cells transfected with empty vector was subtracted from data obtained from cells transfected with NMDA receptor subunits.

### Electrophysiological recording

#### Whole-cell recordings

Experiments were performed 18–36 h after the end of transfection on cultured HEK293T cells transfected with vectors containing hGluN1/hGluN2B/GFP. Whole-cell voltage clamp current recordings were performed at room temperature (21–25°C) at a holding potential of −60 mV using a patch-clamp amplifier (Axopatch 200B; Molecular Devices, Sunnyvale, CA, USA) after a capacitance and series resistance (<10 MΩ) compensation of 80–90%. Data were collected (low-pass filtered at 2 kHz and sampled at 10 kHz) and analyzed using pClamp 10 (Molecular Devices).

Patch pipettes (3–5 MΩ) were filled with an intracellular solution (ICS) containing (in mM): 120 gluconic acid, 15 CsCl, 10 BAPTA, 10 HEPES, 3 MgCl_2_, 1 CaCl_2_, and 2 ATP-Mg salt (pH-adjusted to 7.2 with CsOH) and extracellular solution (ECS) contained the following (in mM): 160 NaCl, 2.5 KCl, 10 HEPES, 10 glucose, 0.2 EDTA, and 0.7 CaCl_2_ (pH-adjusted to 7.3 with NaOH). Glycine (30 μM), an NMDAR co-agonist, was present in the control and test solutions. All steroid solutions were made from freshly prepared 20 mM 20-oxo-pregn-5-en-3β-yl sulfate (PE-S; pregnenolone sulfate), and 5 mM androst-5-en-3β-yl hemisuccinate (AND-hSuc) (Krausova et al., 2018) stock in dimethyl sulfoxide (DMSO). The final dilution to ECS was made at 50°C and followed by 1 min sonication (SONOREX DIGITEC DT 100/H, BADELIN electronic, Berlin, Germany). The same concentration of DMSO was added to all extracellular solutions. Drug applications were made with a microprocessor-controlled multibarrel fast-perfusion system. Changes in the glutamate-induced current in HEK293T cells transfected with hGluN1 and hGluN2B subunits were used to estimate the rate of solution exchange around the cell. Standard ECS was switched to the ECS diluted by water (50%) during glutamate application. The osmolarity was adjusted by sucrose to match the standard ECS. Glutamate (1 mM), glycine (10 μM) and pH was maintained constant in both solutions. The solution exchange rate was estimated to be τ = 12.0 ± 0.9 ms (*n* = 6) for the dish attached cells and τ ~200 μs for open tip pipette (Vyklicky et al., 2016).

#### Single-channel recordings

Outside-out patches were pulled from transfected HEK293T cells, and single-channel recordings were performed at a holding potential of −60 mV (−74 mV after liquid junction potential correction) using an Axopatch 200 A amplifier (filtered at 10 kHz with a 4-pole Bessel filter, and sampled at 25 kHz). ECS and ICS were the same as solutions used for the whole-cell recording. Thick-wall borosilicate patch pipettes were pulled, coated with Sylgard 184 (Dow Corning), and fire-polished achieving resistances between 5 and 10 MΩ. Analysis of single-channel currents was performed using pClamp 10. Recordings were filtered at 2 kHz with an 8-pole Bessel filter. Opening and closing transitions were detected using a 50% threshold criterion. Openings briefer than 446 μs (2.5 × filter rise time) were excluded from the analysis (Colquhoun and Sakmann, 1985).

### Data analysis

#### Agonist dose-response analysis

Normalized amplitudes *(I)* were fit to the following logistic equation:

(1) $$\begin{array}{l} I=1/\left(1+{\left(\text{E}{\text{C}}_{50}\left[agonist\right]\right)}^{h}\right), \end{array}$$

where EC_50_ is the concentration of agonist that produces a half-maximal response, [*agonist*] is the glutamate or glycine concentration, and *h* is the apparent Hill coefficient. Dose-response for glutamate was determined from responses made in the presence of 30 μM glycine and dose-response for glycine was determined from responses made in the continuous presence of 1 mM glutamate.

#### Open probability

The channel open probability (*P*_*o*_) was assessed from the kinetics of the MK-801 (1 μM) inhibition of responses to 1 mM glutamate that was fitted to the kinetic model using Gepasi software (version 3.21; Mendes, 1993, 1997; Mendes and Kell, 1998). The glutamate binding steps were not considered because, in the presence of 1 mM glutamate, NMDAR exists with high probability (>99.6% as predicted from calculations using rate constants determined earlier; Cais et al., 2008) only in doubly liganded states with the channel closed (R) or open (O), and/or in a desensitized state (D).

$$\begin{array}{l} D\overset{k_{r}}{\underset{k_{d}}{⇌}}R\overset{k_{o}}{\underset{k_{c}}{⇌}}O\overset{k_{b}}{\underset{k_{u}}{⇌}}B \end{array}$$

B stands for MK-801 blocked state. The fitting procedure consisted of two steps (Turecek et al., 2004). In the first step, the response induced by 1 mM glutamate in HEK293T cells transfected with the WT or mutated hGluN1/hGluN2B receptors was analyzed for the peak (*I*_*P*_) and steady state response (*I*_*SS*_), and onset of desensitization was determined by the single exponential function (τ_*d*_). Desensitization (*D*) and the kinetic constants describing the onset of desensitization (*k*_*d*_) and resensitization (*k*_*r*_) were determined from Equations 2–4:

(2) $$\begin{array}{l} D\;=\;1-\left(I_{SS}/I_{P}\right) \end{array}$$

(3) $$\begin{array}{l} k_{d}=D/\tau_{d} \end{array}$$

(4) $$\begin{array}{l} k_{r}=\left(1-D\right)/\tau_{d} \end{array}$$

In the second step, *k*_*d*_ and *k*_*r*_ were fixed at values obtained from the first step, and the close rate (*k*_*c*_) at an arbitrary value of 200 s^−1^. The NMDAR response recorded in the presence of 1 mM glutamate, and 1 μM MK-801 was fitted to the model while the opening rate (*k*_*o*_) was set as a free parameter. MK-801 blocking rate (*k*_*b*_) was set to 25 μM^−1^ s^−1^ (Huettner and Bean, 1988; Jahr, 1992; Rosenmund et al., 1995). The binding of MK-801 was virtually irreversible over the time course of the experiment for the WT and the mutated receptors, with the exception of hGluN1/hGluN2B(N615I) (Supplementary Figure S1). In the case of hGluN1/hGluN2B(N615I) receptors, the onset of MK-801 inhibition was fitted to the model where the MK-801 unblocking rate (*k*_*u*_) was set as a free parameter. Microscopic open probability (*P*_*o*_) was calculated as:

(5) $$\begin{array}{l} P_{o}=100\times \;k_{o}/\left(k_{o}+k_{c}\right) \end{array}$$

#### Voltage dependence of the Mg^2+^ effect

The *I–V* relation of the Mg^2+^ inhibitory effect was fitted to the Boltzmann function equation of the form:

(6) $$\begin{array}{l} I\left(V\right)=ag_{0}\left(V-V_{\text{rev}}\right)/\left(a+\left[{\text{Mg}}^{2+}\right]e^{-bV}\right) \end{array}$$

in which *g*_0_ is the estimated conductance of the NMDAR whole-cell response in the absence of extracellular Mg^2+^, *V*_rev_ is the reversal potential of NMDA-induced current, and *a, b* are parameters with the following interpretation:

(7) $$\begin{array}{l} a=K_{\text{d}}e^{bV} \end{array}$$

where *K*_d_ represents the apparent dissociation constant for Mg^2+^ binding to the NMDAR at a given membrane potential (*V*) and

(8) $$\begin{array}{l} b=2\delta F/\left(RT\right) \end{array}$$

where δ indicates the apparent electrical distance of the Mg^2+^ binding site from the outside of the membrane and *F, R*, and *T* have their standard thermodynamic meanings (Abdrachmanova et al., 2002). Unless otherwise noted, recordings were performed at a holding potential of −60 mV (values of the holding potential were corrected for the liquid junction potential, 14 mV).

#### Analysis of the steroid effect

The relative degree of steroid-induced potentiation determined for different steroid doses in individual HEK293T cells was fit to the following logistic equation:

(9) $$I=I_{\max}/\left(1+{\left({\text{EC}}_{50}/[steroid]\right)}^{h}\right)$$

where *I*_*max*_ is the maximal value of potentiation, EC_50_ is the concentration of the steroid that produces half-maximal potentiation of glutamate induced current, [*steroid*] is the steroid concentration and *h* is the apparent Hill coefficient.

### Molecular modeling

#### Homology modeling

We have used our recently refined all-atom homology model of rat NMDAR based on available crystal structures as templates (PDB IDs-4tll, 4tlm, and 4pe5) (Karakas and Furukawa, 2014; Lee et al., 2014). The receptor in these crystal structures contains various sequence modifications (deletions, substitutions, and introduction of disulfide bridges) in order to stabilize the interaction between amino-terminal domains as well as in the TMD region. The homologous sequences were aligned using MUSCLE and visually inspected within Unipro UGENE program. The all-atom model, including the residues missing in the template structures, was prepared using the automodel function of the MODELLER 9v14 suite of programs (Sali and Blundell, 1993; Webb and Sali, 2014), including symmetry restraints for the Cα atoms of corresponding pairs of subunits. We have further created a WT receptor model with MODELLER, using the rat NMDAR model as a template. This NMDAR model quite likely reflects liganded receptor with the channel in the closed state prior to opening.

#### Modeling the effect of rare mutations

Amino acid substitutions within the TMD were introduced using MODELLER. The WT model and each of 11 mutated NMDAR models were subjected to a 10 ns molecular dynamics (MD) simulation. The parameters of implicit solvation/lipid membrane model (EEF1/IMM1) (Lazaridis, 2003) simulations were assigned using the web-based graphical user interface CHARMM-GUI (Jo et al., 2008), and the Langevin MD simulation with 2 fs time step was performed using the CHARMM (Brooks et al., 2009) MD package version c41b1.

#### Visualization of structures

Graphical representation and analysis of residues surrounding selected regions were performed using PyMOL version 1.8.3.

### Statistical analysis

All data are expressed as mean ± SEM. Group differences were analyzed using a one-way ANOVA followed by multiple comparison procedures, and Student's *t*-test or Mann-Whitney Rank Sum Test was used for comparison between experimental groups using the statistical software package SigmaStat 3.5 (Systat Software Inc., San Jose, CA, USA).

## Results

### Rare variants in the TMD reduce surface NMDAR expression

**Figure 1:**
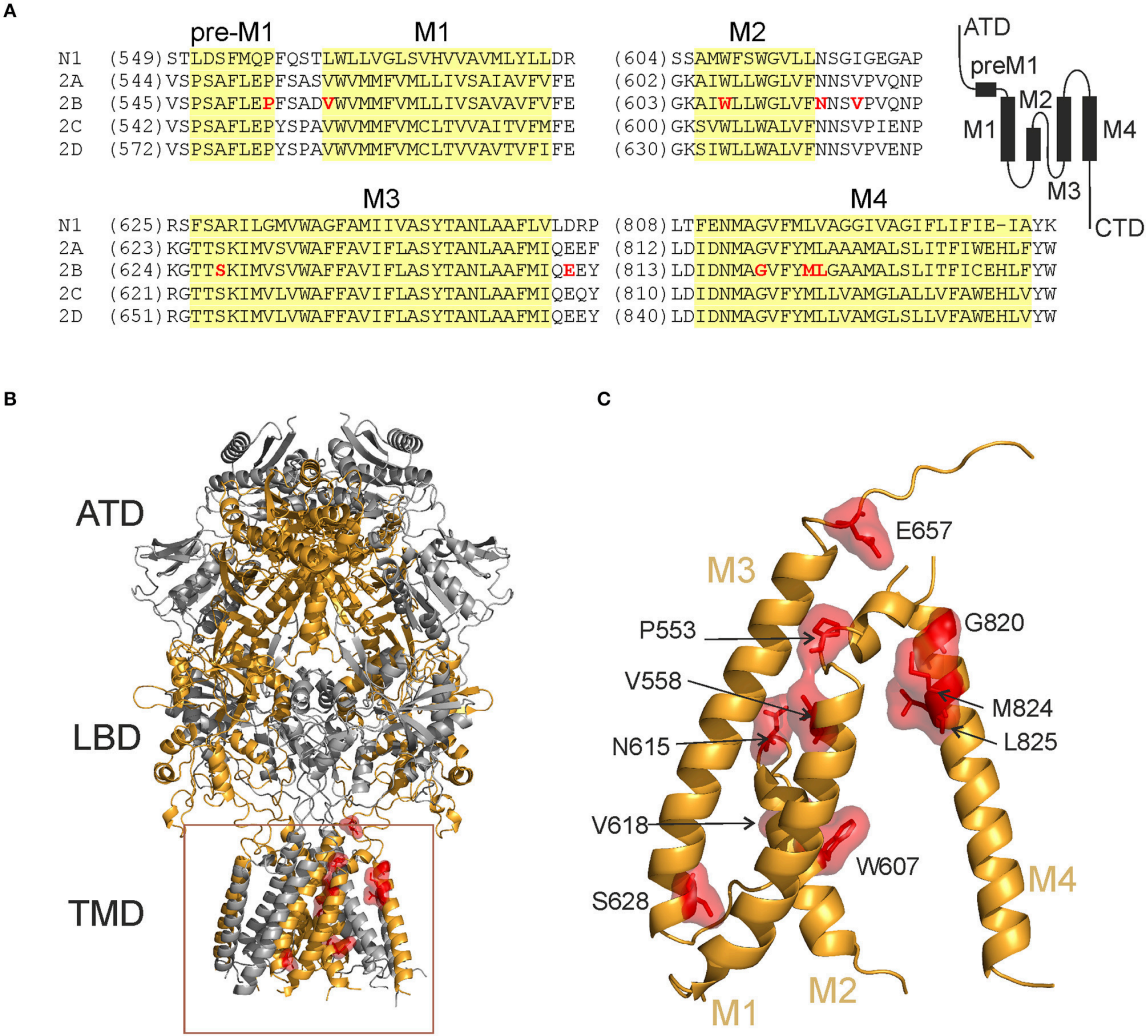
Locations of the hGluN2B rare mutations. **(A)** Domain architecture of NMDARs and protein amino acid sequence alignment showing pre-M1, M1, M2, M3, and M4 regions across NMDAR subunits. Membrane segments are indicated in yellow. **(B,C)** Ribbon structure of a homology model of the hGluN1/hGluN2B receptor (Karakas and Furukawa, 2014; Lee et al., 2014) (hGluN1 in gray; hGluN2B in orange). Selected residues representing amino acids that were altered in human patients are highlighted (in red). ATD, amino-terminal domain; LBD, ligand-binding domain; TMD, transmembrane domain; M1, M3, and M4, transmembrane helices, and M2, re-entrant pore loop.

Recently, we elucidated the effects of different NMDAR mutations in an effort to identify sites of action for inhibitory steroids (Vyklicky et al., 2015). A secondary result of these experiments was the finding that mutations in the TMD and adjacent regions often dysregulated the NMDAR function. We therefore screened available databases (https://www.ncbi.nlm.nih.gov/clinvar/) and selected 11 *de novo* mutations (P553L; V558I; W607C; N615I; V618G; S628F; E657G; G820E; G820A; M824R; L825V) located in the pre-M1, M1, M2, M3, and M4 membrane regions of the hGluN2B subunit (Figure 1, Table 1).

**Table 1:** Selected *de novo GRIN2B* variants and their phenotypic characteristics.

**GluN2B mut**.	**Genotype**	**Phenotype**	**Age of onset**	**Source**
P553L	c.1658C>T	ID, hypotonia	Early postnatal	de Ligt et al., 2012
V558I	c.1672G>A	ID	-	Hamdan et al., 2014; Lelieveld et al., 2016
W607C	c.1821G>T	ID, DD, dysmorphic features	-	Yavarna et al., 2015
N615I	c.1844A>T	WS, ID	7 weeks	Lemke et al., 2014
V618G	c.1853T>G	ID, WS, Epi-encephalopathy	4 months	Lemke et al., 2014
S628F	c.1883C>T	ID, DD, Epi-encephalopathy	-	Platzer et al., 2017
E657G	c.1970A>G	ID, DD	-	Platzer et al., 2017
G820E	c.2459G>A	ID, microcephaly	Early postnatal	Hamdan et al., 2014
G820A	c.2459G>C	ID, DD, DMD, ES, GVL, ASD	-	Platzer et al., 2017
M824R	c.2471T>G	ID, DD, microcephaly, Rett-like picture, Epi activity on EEG	2 months	Zhu et al., 2015
L825V	c.2473T>G	ASD	-	Awadalla et al., 2010; Swanger et al., 2016

*ID, intellectual disability; DD, developmental delay; WS, West Syndrome; DMD, Dyskinetic Movement Disorder; ES, epileptic spasms; GVL, generalized cerebral volume loss; ASD, Autism Spectrum Disorder; Epi, epilepsy and/or seizures, infantile spasms*.

There is no or incomplete information available about the effects of these missense mutations on NMDAR function. We therefore evaluated the amplitude of glutamate-induced responses, relative cell-surface expression, agonist potency, receptor desensitization, relative *P*_*o*_, and pharmacological properties of each of these variants as an initial step in understanding the potential role NMDAR channelopathy may play in the pathophysiology of neurological diseases. HEK293T cells transfected with WT receptors responded to glutamate (1 mM; applied in the continuous presence of 30 μM glycine) with peak currents of 66 pA/pF. Receptors with mutated hGluN2B subunit either did not respond to glutamate application (currents <5 pA) (P553L; S628F; G820E; M824R) or responded with significantly reduced amplitudes (W607C; N615I; V618G; E657G; G820A). The amplitude of responses was not significantly different for hGluN1/hGluN2B(V558I) and hGluN1/hGluN2B(L825V) receptors (Supplementary Figure S2). The differences in the peak amplitude normalized with respect to the cell capacitance must be interpreted cautiously since this is influenced by factors that we cannot fully control. We have hypothesized that reduced protein levels and/or altered receptor function may underlie reduced responses to 1 mM glutamate in HEK293T cells expressing mutated NMDARs.

**Figure 2:**
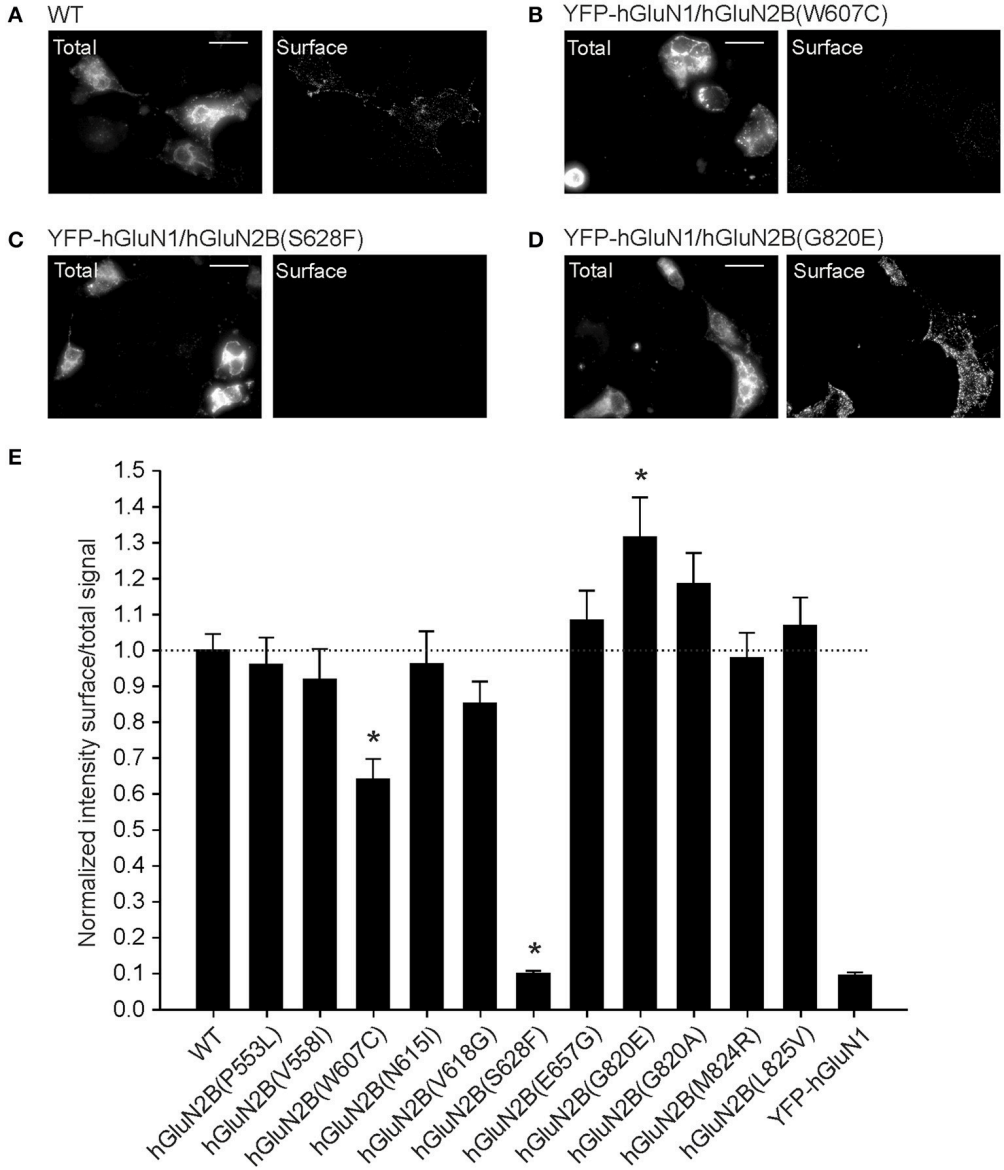
Mutations in the TMD of hGluN2B subunit affect the NMDAR surface expression. **(A–D)** Representative images of the total (left panel) and surface (right panel) pools of YFP-hGluN1 expressed in COS-7 cells. COS-7 cells were transfected with WT **(A)**, YFP-hGluN1/hGluN2B(W607C) **(B)**, YFP-hGluN1/hGluN2B(S628F) **(C)**, and YFP-hGluN1/hGluN2B(G820E) **(D)** receptors. A scale bar in **(A–D)** represents 20 μm. **(E)** An immunofluorescence microscopy was used to determine relative surface-to-total NMDAR levels in COS-7 cells transfected with only YFP-hGluN1 subunit for control (YFP-hGluN1) or both the YFP-hGluN1 and hGluN2B (WT) or mutated hGluN2B subunit. Data show mean ± SEM; *n* = 35 in three experiments. ^*^p < 0.05 relative to WT, one-way ANOVA (Multiple comparisons vs. WT-Dunnett's method). For original images see (Supplementary Figures S5–S12).

Next, we used immunofluorescence microscopy to determine the surface and total protein levels of the mutated NMDARs. First, we generated a gene encoding for YFP-hGluN1 (see Methods). In the second step, COS-7 cells were transfected with genes encoding for YFP-hGluN1 and WT or mutated hGluN2B subunits. Quantitative analysis of images indicated that the ratio of surface-to-total NMDAR expression was significantly reduced for YFP-hGluN1/hGluN2B(W607C) receptors compared to WT receptors (Figures 2A,B), and the surface expression of YFP-hGluN1/hGluN2B(S628F) receptors (Figure 2C) was quite likely completely lost since the ratio of the surface-to-total receptor levels was the same as that determined for COS-7 cells transfected with YFP-hGluN1 subunit only (Horak et al., 2008; Kaniakova et al., 2012). In addition, the microscopy assay in cells expressing YFP-hGluN1/hGluN2B(G820E) receptors indicated increased surface expression compared to the WT receptors (Figure 2D). Figure 2E shows relative surface expression of the WT and mutated hGluN2B subunits. YFP-hGluN1 is not surface expressed unless it is co-expressed with the hGluN2B subunit (Okabe et al., 1999; Standley et al., 2000). In addition, surface-to-total expression of the WT and YFP-hGluN1/hGluN2B(W607C; S628F; and G820E) receptors was assessed using immunofluorescence microscopy on HEK293T cells and a quantitative expression assay in COS-7 cells (see Methods). The data obtained using these methods confirmed a significant decrease in the surface expression of YFP-hGluN1/hGluN2B(S628F) receptors compared to the WT and increase in the surface expression of YFP-hGluN1/hGluN2B(G820E) receptors, however, differences in the surface expression of YFP-hGluN1/hGluN2B(W607C) receptors were significant only for data gained by immunofluorescence microscopy on HEK293T cells (Supplementary Figure S3).

Thus, it is possible to explain the insensitivity of hGluN1/hGluN2B(S628F)-transfected cells to glutamate by virtually no surface expression of the receptors. In contrast, insensitivity or reduced sensitivity of cells transfected with hGluN1 and hGluN2B(P553L; W607C; N615I; V618G; E657G; G820E; G820A; or M824R) subunits contrasts with normal or increased surface expression and indicates impaired receptor function.

### Mutations related to human neurological diseases decrease agonist potency

**Figure 3:**
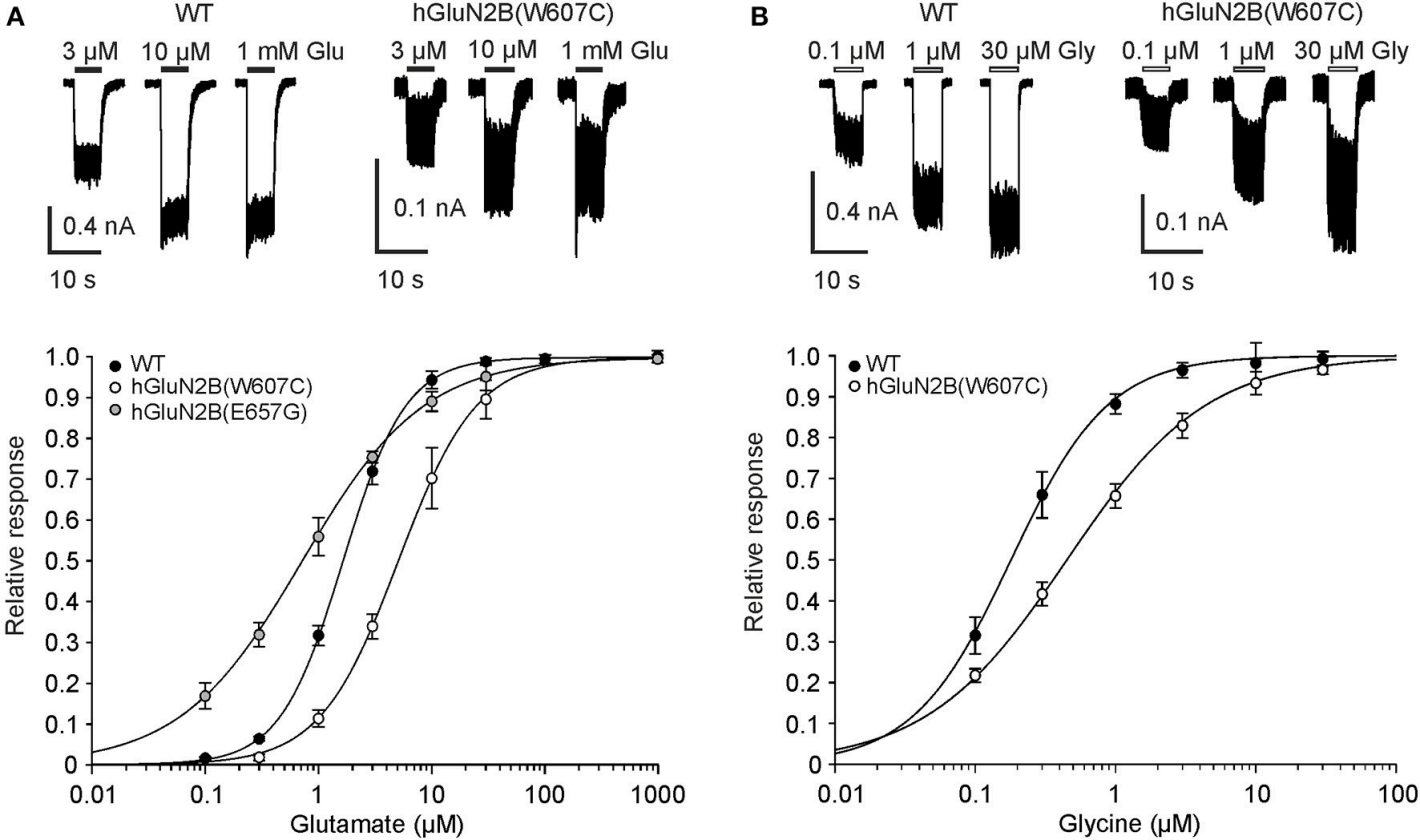
hGluN2B rare variants alter glutamate and glycine potency. **(A)** Representative recordings of responses to 3, 10, and 1,000 μM glutamate applications made in the presence of 30 μM glycine are shown for HEK293T cells expressing WT and hGluN1/hGluN2B(W607C) receptors. Peak concentration-response relationship for glutamate activation of WT, hGluN1/hGluN2B(W607C; and E657G) receptors. **(B)** Representative recordings of responses to 0.1, 1, and 30 μM glycine applications made in the presence of 1 mM glutamate are shown for HEK293T cells expressing WT and hGluN1/hGluN2B(W607C) receptors. Peak concentration-response relationship for glycine activation of WT and hGluN1/hGluN2B(W607C) receptors. Data points in **(A,B)** are averaged values of relative currents form at least five independent measurements. Error bars represent SEM. Values of glutamate and glycine EC_50_, *h* and *n* are listed in Table 2.

Seven mutations in the TMD responded to 1 mM glutamate and 30 μM glycine with currents that allowed reliable dose-response analysis of the agonist-induced responses. This analysis indicated that glutamate is 3.2- and 1.7-fold less potent at hGluN1/hGluN2B(W607C; and N615I) receptors and 2.3-fold more potent at hGluN1/hGluN2B(E657G) receptors than at the WT receptors (Figure 3A, Table 2). Similar analysis of glycine dose-response suggested that glycine is 2.0- and 1.8-fold less potent at hGluN1/hGluN2B(W607C; and E657G) receptors than at the WT receptors (Figure 3B, Table 2). Altered glutamate potency in hGluN1/hGluN2B(E657G) receptors and altered glycine potency in hGluN1/hGluN2B(W607C; and E657G) receptors was accompanied by a diminished value of the Hill coefficient indicating on altered degree of cooperativity of the agonists binding to the receptor (Weiss, 1997).

**Table 2:** Summary of the agonist potency data.

**Mutation**	**Glutamate**			**Glycine**		
	**EC_50_ (μM)**	***h***	***n***	**EC_50_ (μM)**	***h***	***n***
hGluN2B(WT)	1.6 ± 0.1	1.4 ± 0.1	15	0.22 ± 0.03	1.45 ± 0.11	9
hGluN2B(V558I)	1.6 ± 0.3	1.5 ± 0.2	5	0.22 ± 0.05	1.14 ± 0.05	5
hGluN2B(W607C)	5.1 ± 0.4^*^	1.3 ± 0.2	5	0.45 ± 0.08^*^	0.87 ± 0.05^*^	7
hGluN2B(N615I)	2.7 ± 0.2^*^	1.2 ± 0.1	5	0.31 ± 0.02	1.41 ± 0.10	5
hGluN2B(V618G)	1.7 ± 0.2	1.2 ± 0.1	5	0.28 ± 0.03	1.43 ± 0.09	5
hGluN2B(E657G)	0.7 ± 0.1^*^	0.8 ± 0.1^*^	9	0.39 ± 0.08^*^	0.97 ± 0.10^*^	5
hGluN2B(G820A)	1.5 ± 0.3	1.0 ± 0.2	4	0.20 ± 0.09	1.15 ± 0.05	3
hGluN2B(L825V)	1.5 ± 0.1	1.1 ± 0.1	9	0.16 ± 0.01	1.26 ± 0.12	5

*The data are expressed as mean ± SEM*.

*n is the number of cells recorded from*.

* *p < 0.05; statistical tests on potency were performed on logEC_50_ and logHill compared to the corresponding WT; one-way ANOVA, Tukey post-hoc Dunnett's method*.

### Mutations in the TMD alter receptor desensitization and decrease channel open probability

**Figure 4:**
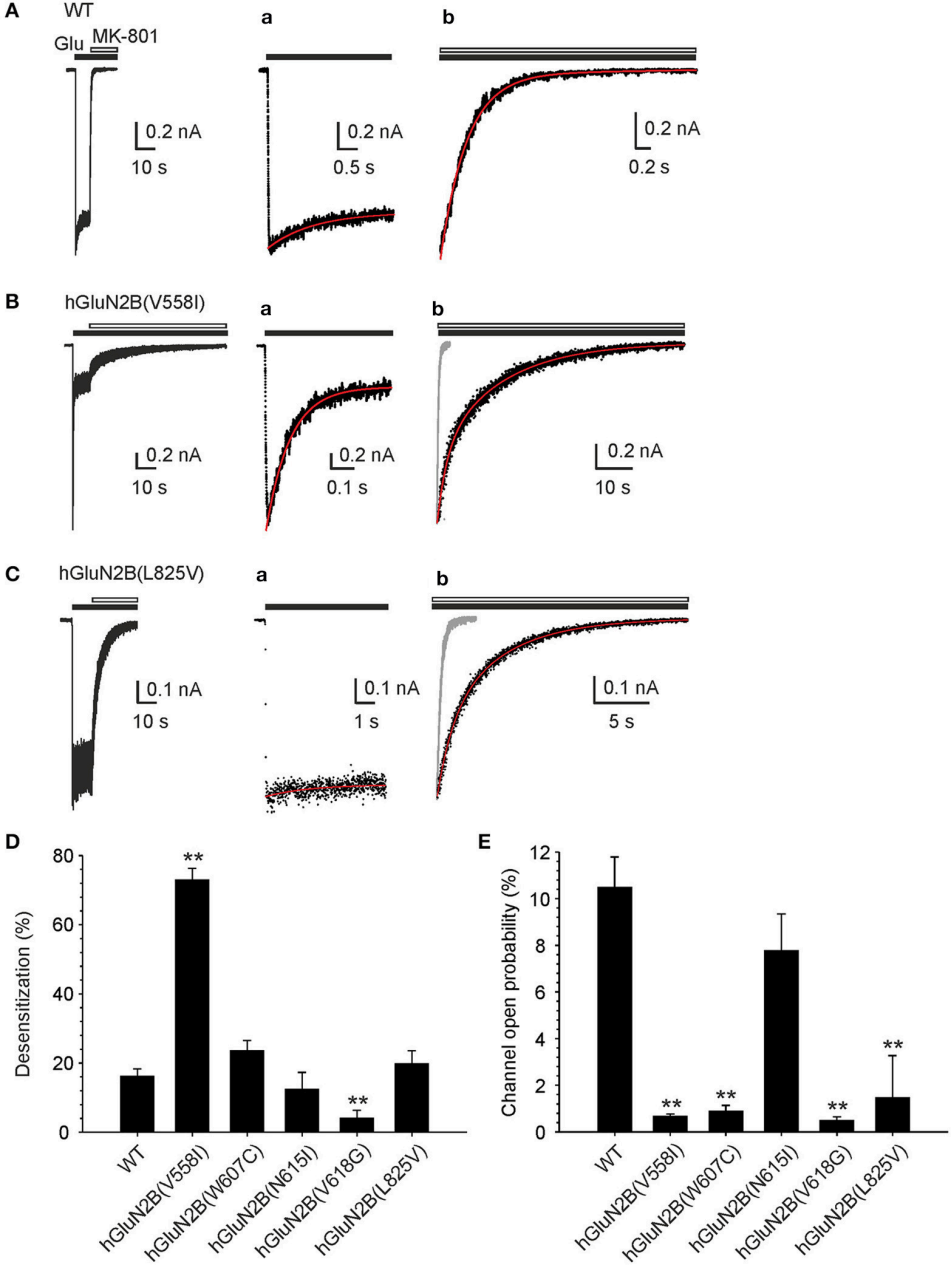
Mutations in the TMD alter receptor desensitization and *P*_*o*_. **(A-C)** Representative currents induced in WT, hGluN1/hGluN2B(V558I; and L825V) receptors by fast application of 1 mM glutamate (in the continuous presence of 30 μM glycine) and their inhibition by MK-801 (1 μM). **(Aa-Ca)** Show the response to glutamate at an expanded time scale. Desensitization of WT receptors was best described by rate constants *k*_*d*_ = 0.17 s^−1^ and *k*_*r*_ = 0.69 s^−1^; desensitization of hGluN1/hGluN2B(V558I) receptors by *k*_*d*_ = 4.06 s^−1^ and *k*_*r*_ = 1.12 s^−1^, and desensitization of hGluN1/hGluN2B(L825V) receptors by *k*_*d*_ = 0.11 s^−1^ and k_r_ = 0.83 s^−1^ (indicated by a superimposed red trace). **(Ab-Cb)** Show the onset of MK-801 inhibition on an expanded time scale. The time course of the onset of MK-801 (gray response in Bb and Cb) inhibition was best described by *P*_*o*_ = 12.2% for WT receptors, *P*_*o*_ = 0.61% for hGluN1/hGluN2B(V558I) receptors, and *P*_*o*_ = 1.16% for hGluN1/hGluN2B(L825V) receptors (indicated by a superimposed red trace) (see Methods section for details of the analysis procedure). Graphs show the summary of mean desensitization **(D)** and *P*_*o*_ **(E)** ± SEM (*n*) for WT and mutated receptors. The differences in the mean values of desensitization and *P*_*o*_ determined for the WT and mutated receptors was statistically significant; Kruskal-Wallis one-way ANOVA on Ranks, *p* < 0.001; subsequent Mann-Whitney Rank Sum Test or unpaired *t*-test was used to assess the significance compared to the WT, ^*^p = 0.001–0.05, ^**^p < 0.001.

To evaluate the effects of mutations in the TMD on NMDAR desensitization, the receptors were activated by a saturating concentration of glutamate (1 mM) in the continuous presence of glycine (30 μM). The analysis of the desensitization—a time-dependent decline of responses (see Equation 2 for definition)—indicated that WT receptors desensitized on average by 16.1% (Figure 4A). The desensitization was significantly increased to 72.9% in hGluN1/hGluN2B(V558I) receptors (Figure 4B) and was not significantly changed in hGluN1/hGluN2B(L825V) receptors (Figure 4C); by contrast, hGluN1/hGluN2B(V618G) receptors desensitized only by 4.1% (Figure 4D).

The time course of the desensitization onset of responses induced in the WT and hGluN1/hGluN2B(V558I; W607C; N615I; and L825V) receptors was next analyzed and desensitization (*k*_*d*_) and (*k*_*r*_) resensitization rate constants were determined (see Equations 2-4). The desensitization observed in the WT receptor responses was characterized by *k*_*d*_ = 0.24 ± 0.08 s^−1^ (*n* = 10). The value of *k*_*d*_ was significantly increased in hGluN1/hGluN2B[V558I (3.55 ± 0.46 s^−1^; *n* = 7); and W607C (1.59 ± 0.54 s^−1^; *n* = 4)] receptor responses (one-way ANOVA, *p* < 0.001; followed by multiple comparisons of the rate constant determined in the mutated receptors vs. the WT; Dunnett's method, *p* < 0.050). The values of the *k*_*d*_ determined in hGluN1/hGluN2B[N615I (0.48 ± 0.14 s^−1^; *n* = 4); and L825V (0.43 ± 0.16 s^−1^; *n* = 6)] receptor responses was not significantly different from that determined in WT. WT receptor responses were characterized by *k*_*r*_ = 1.06 ± 0.17 s^−1^ (*n* = 10). The value of the *k*_*r*_ was significantly increased in hGluN1/hGluN2B[W607C (4.79 ± 1.22 s^−1^; *n* = 4); and N615I (2.61 ± 0.51 s^−1^; *n* = 4)] receptor responses (one-way ANOVA, *p* < 0.001; followed by multiple comparisons of the rate constant determined in the mutated receptors vs. the WT; Dunnett's method, *p* < 0.050). The values of the *k*_*r*_ determined in hGluN1/hGluN2B[V558I (1.29 ± 0.28 s^−1^; *n* = 7); and L825V (1.47 ± 0.20 s^−1^; *n* = 6)] receptors was not significantly different from that determined in WT receptors. Low amplitude of the hGluN1/hGluN2B(V618G) receptor responses precluded detailed analysis of the rate constants characterizing receptor desensitization.

To evaluate the effects of mutations in the TMD on *P*_*o*_, the WT and mutated receptors were activated by a saturating concentration of glutamate (1 mM) and the rate of channel block by 1 μM MK-801 was fitted to the kinetic model and the *P*_*o*_ determined (see Methods and Equation 5). Using this approach, the *P*_*o*_ of the human WT receptors was determined to be 10.5 ± 1.3% (*n* = 10) (Figure 4A). This value is not different from the *P*_*o*_ determined from the analysis of single-channel of activity induced by 1 mM glutamate in cell-attached patches (*P*_*o*_ = 10.6 ± 2.9% (*n* = 12); unpaired *t*-test, *p* = 0.977) (see Supplementary Figure S4) and, in addition, it agrees well with that determined for rat GluN1/GluN2B receptors (Chen et al., 1999).

Superimposed traces indicate that in hGluN1/hGluN2B(V558I; and L825V) receptors, the onset of MK-801 inhibition was decelerated compared to WT receptors (Figures 4A–C). The results of the analysis indicated that the *P*_*o*_ decreased from 10.5% in WT receptors to 0.66% in hGluN1/hGluN2B(V558I) and 1.47% in hGluN1/hGluN2B(L825V) receptors. Similar decrease in *P*_*o*_ was observed in hGluN1/hGluN2B(W607C; and V618G) receptors (Figure 4E). These data indicate that *de novo* mutations in the TMD alter receptor desensitization and *P*_*o*_.

### Mutations in the channel pore alter Mg^2+^ inhibition and single-channel amplitude

**Figure 5:**
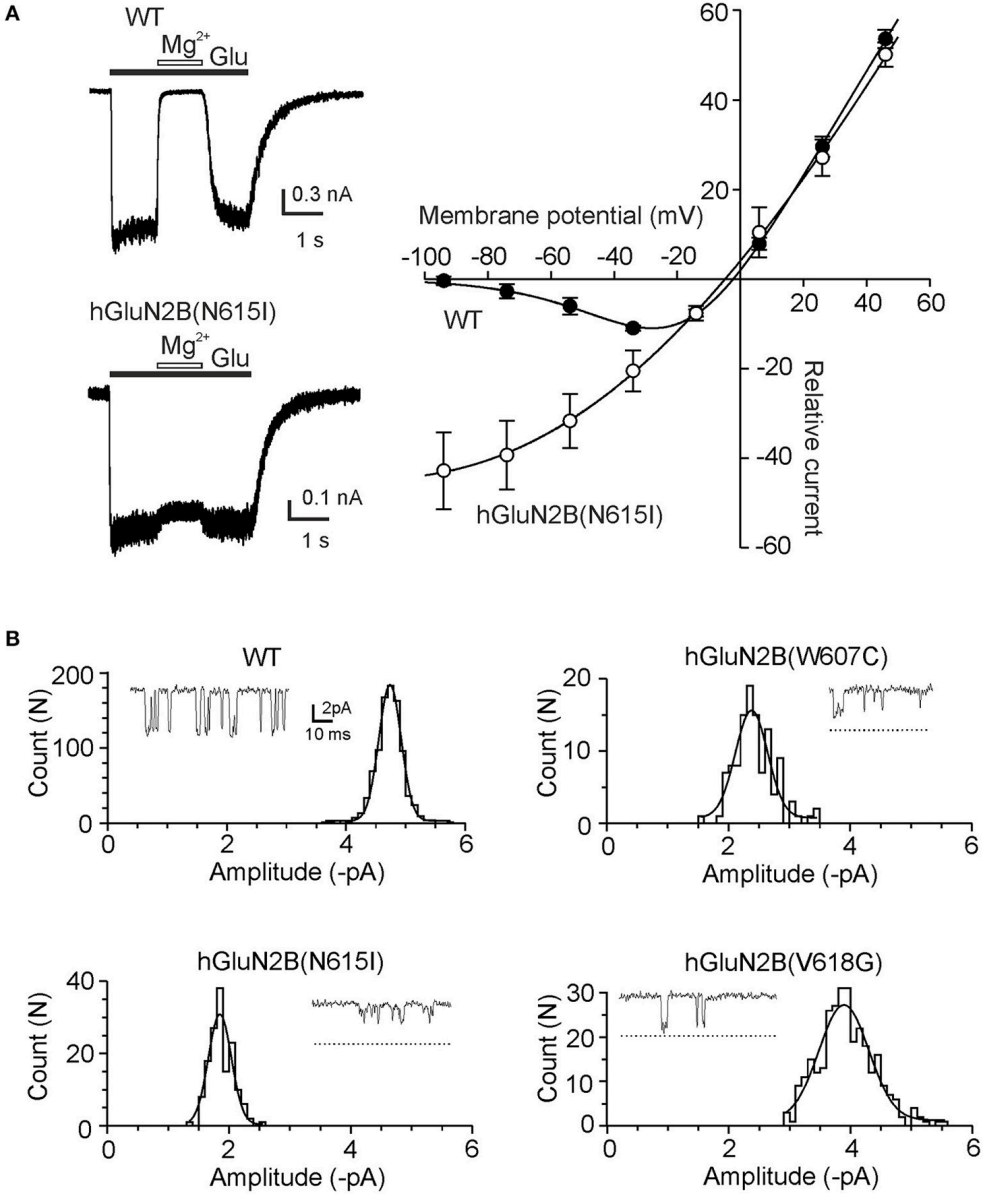
Mutations in the channel pore alter ion permeation. **(A)** Effect of Mg^2+^ on WT and hGluN1/GluN2B(N615I) receptor responses. Whole-cell current responses were induced by 1 mM glutamate in the absence and presence of 1 mM Mg^2+^ at a holding potential of −60 mV. *I-V* relations without Mg^2+^ were fit by a linear equation and normalized to have a slope of 1. Corresponding responses recorded in the presence of Mg^2+^ were normalized and fitted to the Boltzmann equation (Equations 6–8). **(B)** Examples of single-channel openings induced in WT, hGluN1/hGluN2B(W607C; N615I; and V618G) receptors by 1 mM glutamate. Dashed lines show where the current levels identified from the fit to the amplitude distribution for the WT would fall. For individual patches the distribution is shown fitted with a single Gaussian component. Glutamate-induced single-channel currents had mean amplitude: −4.7 pA (WT), −2.4 pA [hGluN1/hGluN2B(W607C)], −1.9 pA [hGluN1/hGluN2B(N615I)], and −3.9 pA [hGluN1/hGluN2B(V618G)].

Structural data indicate that residues hGluN2B(W607; N615; and V618) are part of the NMDAR channel pore. A functionally important feature of NMDARs is the voltage-dependent inhibition by extracellular Mg^2+^, and several residues located within the ion channel pore have been shown to alter Mg^2+^ sensitivity (Kuner et al., 1996; Kupper et al., 1996; Williams et al., 1998). Fitting the current-voltage relation of WT receptor responses recorded in the presence of 1 mM Mg^2+^ to the Boltzmann function (Equations 6-8) revealed that the affinity for Mg^2+^ in the absence of an electric field (K_d(0__mV)_) was 5.9 ± 2.3 mM and δ = 0.45 ± 0.07 (*n* = 5) (Figure 5A). hGluN1/GluN2B(N615I) receptor responses were only slightly affected by 1 mM Mg^2+^, and the fit to the Boltzmann function provided unreliable values for both K_d(0__mV)_ and δ (Figure 5A). The responses induced in hGluN1/hGluN2B(W607C; and V618G) receptors were small, precluding detailed analysis of the current-voltage relation. Based on the relative inhibitory effect of 1 mM Mg^2+^ at a holding potential of −60 mV, which decreased from 97 ± 2% (*n* = 5) in WT to 35 ± 3% (*n* = 6) in hGluN1/hGluN2B(W607C), we infer that Mg^2+^ binding within these receptor channels is also altered. The responses induced by hGluN1/hGluN2B(V618G) were small, precluding any reliable analysis of the Mg^2+^ effect. These results agree well with reduced Mg^2+^ block observed in mutated receptors (Lemke et al., 2014).

Next, we aimed to characterize the effect of mutations on the ion permeation employing single-channel recording from outside-out patches. Figure 5B shows records of channel activity in outside-out patches recorded in response to the application of 1 mM glutamate in the presence of Mg^2+^-free extracellular solution at a holding potential of −60 mV (−74 mV after junction potential correction). The mean amplitude of the principal single-channel current was decreased from −4.5 ± 0.1 pA (*n* = 6) in the WT receptors to −2.4 ± 0.1 pA (*n* = 5) for hGluN1/hGluN2B(W607C) receptor channels (*p* < 0.001, compared to WT), −1.8 ± 0.1 pA (*n* = 5) for hGluN1/hGluN2B(N615I) receptor channels (*p* < 0.001, compared to WT), and −3.6 ± 0.1 pA (*n* = 5) for hGluN1/hGluN2B(V618G) receptor channels (*p* < 0.001, compared to WT). These data indicate that mutations in the NMDAR channel pore have opposing effects –an increase in responses due to the reduced Mg^2+^ inhibitory effect and a reduction of responses due to the decrease in the single-channel amplitude; the overall effect will be a function of the membrane potential.

### GluN2B(L825V) mutation enhances sensitivity to endogenous and synthetic steroids

**Figure 6:**
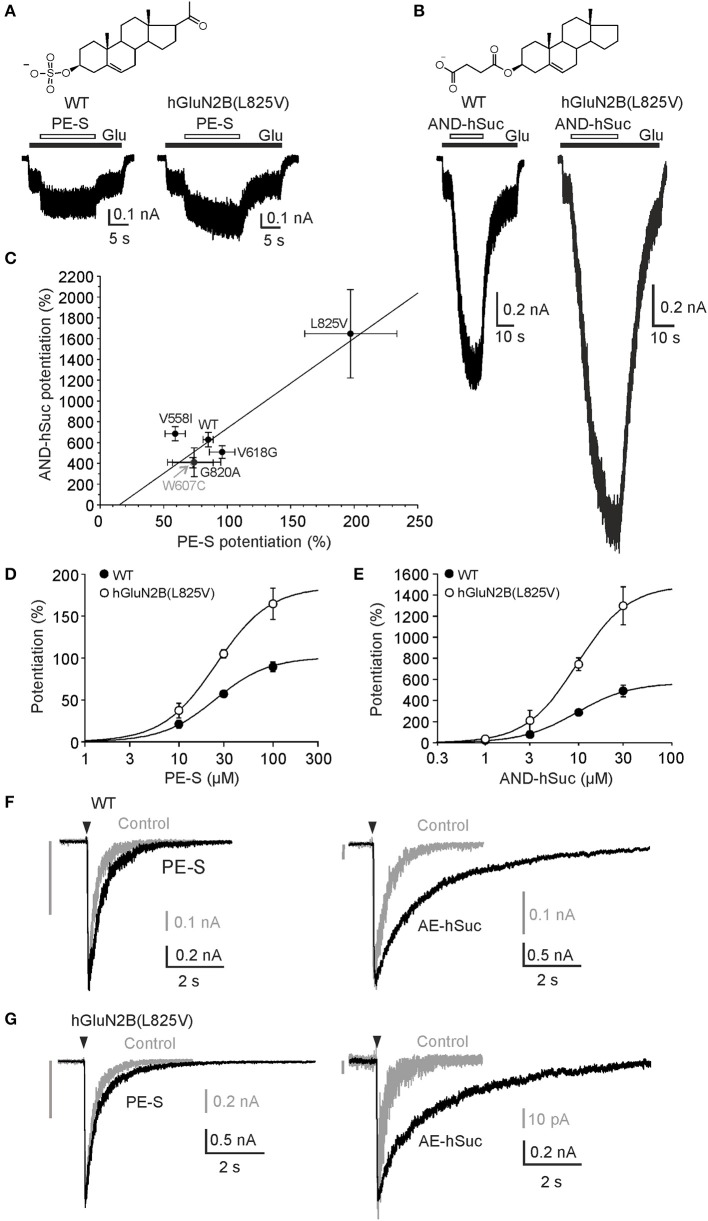
hGluN1/hGluN2B(L825V) has an enhanced sensitivity to steroids. **(A)** Representative responses are shown for WT and hGluN1/hGluN2B(L825V) receptors. PE-S (100 μM) **(A)** and AND-hSuc (30 μM) **(B)** potentiated the WT receptor responses induced by co-application with 1 μM glutamate in the continuous presence of 30 μM glycine to a different extent (by 85% and 575%, respectively). Both steroids potentiated responses induced in hGluN1/hGluN2B(L825V) receptors ~2-fold more than in WT receptors. Structure of PE-S **(A)** and AND-hSuc **(B)**. **(C)** Graph shows relative PE-S (100 μM) potentiation ± SEM (*n* = 5–31) plotted vs. that induced by AND-hSuc (30 μM) ± SEM in the WT and in mutated receptors. Data were fit by a linear regression (Correlation coefficient r = 0.928; *p* = 0.00764). Concentration dependency for PE-S **(D)** and AND-hSuc **(E)** induced potentiation of WT and hGluN1/hGluN2B(L825V) receptors activated by 1 μM glutamate in the continuous presence of 30 μM glycine. Parameters of the fit (see Equation 9) were for the WT receptors: PE-S induced relative maximal potentiation *I*_*max*_ = 101 ± 9%; EC_50_ = 24.3 ± 1.8 μM with *h* = 1.54 ± 0.08 (*n* = 7); AND-hSuc *I*_*max*_ = 570 ± 57%; EC_50_ = 9.5 ± 1.0 μM; *h* = 1.50 ± 0.16 (*n* = 6). In hGluN1/hGluN2B(L825V) receptors: PE-S induced *I*_*max*_ = 185 ± 50% (*p* = 0.030 compared to WT); EC_50_ = 24.8 ± 1.2 μM (*p* = 0.842 compared to WT) with *h* = 1.52 ± 0.12 (*n* = 5); AND-hSuc *I*_*max*_ = 1492 ± 514% (*p* = 0.030 compared to WT); EC_50_ = 9.6 ± 2.3 μM (*p* = 0.961 compared to WT) with *h* = 1.58 ± 0.36 (*n* = 6). **(F,G)** Normalized representative current responses of WT and hGluN1/hGluN2B(L825V) receptors induced by brief (5 ms) application of 1 mM glutamate made in the ECS (Control, gray) and in the presence of PE-S (100 μM) or AND-hSuc (30 μM) following steroid pre-application for (>30 s). The vertical gray bar indicates the amplitude of the control response at the same amplitude scale as that recorded in the presence of steroid. See Table 3 for values and statistics on the steroid effect.

Endogenous steroids are potent allosteric modulators of NMDAR activity (Korinek et al., 2011). The sensitivity of the mutant receptors to extracellular PE-S, a steroid that has a positive allosteric and subunit-dependent effect at NMDAR (with preference for GluN2A/B subunits) (Wu et al., 1991; Horak et al., 2006), was evaluated by generating dose-response curves for the potentiation of responses to glutamate. In addition, we analyzed the potentiating effect for AND-hSuc, a more potent synthetic PE-S analog (Krausova et al., 2018). Since the effect of steroids with a potentiating effect at NMDAR is disuse-dependent (Horak et al., 2004), a low concentration of glutamate (1 μM) was used in these experiments. Figures 6A,B shows that PE-S and AND-hSuc potentiate both the WT and mutated receptors, although to a different extent. Analysis revealed that GluN1/GluN2B(L825V) receptors were significantly more potentiated by a nearly saturating concentration of PE-S (100 μM) than responses induced in the WT and hGluN1/hGluN2B(V558I; W607C; V618G; and G820A) receptors (Figure 6C; Kruskal-Wallis one-way ANOVA on Ranks, *p* < 0.001; unpaired *t*-test, *p* < 0.001). Similarly to the effect of PE-S, AND-hSuc (30 μM) potentiated glutamate responses induced in the mutated receptors with a higher potency than in the WT (Figure 6C; Kruskal-Wallis one-way ANOVA on Ranks, *p* < 0.001; unpaired *t*-test, *p* < 0.001). GluN1/GluN2B(L825V) receptors were potentiated robustly (1647%) by AND-hSuc. Receptors containing hGluN1 and hGluN2B(V558I; W607C; V618G; or G820A) subunits were potentiated to an extent not significantly different from that determined for the WT receptors (Figure 6C; *p* = 0.117–0.412 for PE-S; *p* = 0.448–0.966 for AND-hSuc). Dose-response analysis of the effect of PE-S and AND-hSuc indicated that the increased steroid potentiation of GluN1/GluN2B(L825V) receptors was attributable to the increase in steroid efficacy rather than potency (Figures 6D,E).

The time course of the NMDAR responses following brief glutamate application has been suggested to mimic the time course of the NMDAR component of the EPSCs (Lester et al., 1990). To evaluate the effects of steroids on the deactivation time course, we measured current responses induced by a brief (~5 ms) application of 1 mM glutamate to HEK293T cells expressing WT or hGluN1/hGluN2B(L825V) receptors. In the WT receptors, PE-S (100 μM) significantly increased the amplitude of synaptic-like responses and slowed the deactivation (Figure 6F, Table 3). The deactivation of hGluN1/hGluN2B(L825V) receptors was not significantly different (*p* = 0.696) from that determined for the WT receptors. This agrees well with the prediction made from the simulation of responses to brief glutamate application to NMDARs that have diminished *P*_*o*_ but unaltered glutamate EC_50_ (Figure 4E, Table 2). In the hGluN1/hGluN2B(L825V) receptors, PE-S (100 μM) significantly increased the amplitude of synaptic-like responses and slowed the deactivation (Figure 6G, Table 3).

**Table 3:** Summary of the pharmacological properties for synaptic-like responses.

**Receptor**		**Control**	**PE-S (100 μM)**	**Control**	**AND-hSuc (30 μM)**
**WT**	Potentiation (%)	–	98.7 ± 11.5 (5)	–	924.5 ± 172.7 (5)
	τ (ms)	307 ± 14 (5) ^†^	499 ± 20 (5)^‡^^*^	370 ± 35 (5)^†^	5,961 ± 542 (5)^†^^*^
	Deceleration	–	1.63 ± 0.06 (5)	–	16.36 ± 1.44 (5)^†^
	Charge transfer	–	3.26 ± 0.30 (5)	–	161.4 ± 20.3 (5)
**hGluN2B(L825V)**	Potentiation (%)	–	140.8 ± 10.8 (5)	–	2,323 ± 121 (5)
	τ (ms)	323 ± 9 (5)^†^	507 ± 28 (5)^‡^^*^	335 ± 29 (5)^†^	2,467 ± 168 (5)^†^^*^
	Deceleration	–	1.57 ± 0.05 (5)	–	7.19 ± 0.59 (5)^†^
	Charge transfer	–	3.79 ± 0.25 (5)	–	179.7 ± 9.1 (5)

*The data are expressed as mean ± SEM (n) is the number of cells recorded from*.

* *p < 0.001 compared to WT; paired t-test*.

† *τ determined from the single exponential fit*.

‡ *τ_w_ determined from the double exponential fit*.

*Deceleration is defined as τ_steroid_/τ_control_*.

*Charge transfer is defined as τ × ((potentiation/100)+1)*.

Next, we estimated the relative charge transfer recorded for synaptic-like responses in the presence of PE-S. The charge transfer was calculated as a product of the response amplitude and deactivation time course, and its value increased 3.3-fold for the WT and 3.8-fold for hGluN1/hGluN2B(L825V) receptors when synaptic-like responses were recorded in the presence of PE-S (100 μM) (Table 3). Even though this augmentation of synaptic-like responses is prominent, it is not sufficient to compensate for a 7-fold diminution of the synaptic-like response of hGluN1/hGluN2B(L825V) receptors due to their decreased *P*_*o*_ (Figure 4E). The increase in the charge transfer was much more robust for synaptic-like responses recorded in the presence of AND-hSuc (30 μM)-its value increased 161-fold for the WT and 180-fold for hGluN1/hGluN2B(L825V) receptors (Figures 6F,G, Table 3). Estimates indicate that the augmentation of synaptic-like responses is more than sufficient to compensate for a 7-fold diminution of the synaptic-like response of hGluN1/hGluN2B(L825V) receptors when we take into an account their decreased *P*_*o*_ (Figure 4E).

Altogether, these results suggest that (i) PE-S and AND-hSuc have a potentiating effect at human WT receptors; (ii) AND-hSuc is a more potent positive allosteric modulator than PE-S; (iii) even though the potentiating effect of steroids at NMDAR is associated with a disuse-dependent mechanism of action, the effect of both PE-S and AND-hSuc was similar at WT and mutated receptors (hGluN1/hGluN2B(V558I; W607G; and V618G) receptors) that have *P*_*o*_ diminished ~10-fold; (iv) hGluN1/hGluN2B(L825V) receptors were potentiated by both steroids more than WT and other mutated receptors; (v) AND-hSuc-induced increase in the charge transfer of synaptic-like responses can compensate for the mutation-induced diminution of *P*_*o*_ in hGluN1/hGluN2B(L825V) receptors; and (vi) an increase in the positive allosteric effect of both steroids at hGluN1/hGluN2B(L825V) receptors is due to an increase in their efficacy rather than potency.

## Discussion

The development of next-generation DNA sequencing has given rise to unique data linking patient variants in *GRIN1* and *GRIN2* genes to neurological and psychiatric disorders. While the list of rare variants expands rapidly, the consequences for receptor biogenesis, its structure, function, and pharmacology have been only partially revealed (Yuan et al., 2014; Swanger et al., 2016; Chen et al., 2017; Ogden et al., 2017; Platzer et al., 2017). In this study, we investigated 11 disease-associated rare variants in the GluN2B TMD and found that dysregulation of NMDARs involves multiple mechanisms - altered surface expression, agonist potency, receptor desensitization, probability of channel opening, single-channel currents, Mg^2+^ sensitivity - and their combination. Through functional assessments, we show that loss-of-function variants can be rectified by a pharmacological approach, using PE-S and its synthetic analog AND-hSuc. Impaired surface expression and/or receptor function have important implications that may help to find links connecting genetic variation to disease as well as to understand the role of NMDAR variants in neurodevelopmental disorders (Mcrae et al., 2017).

### Mechanisms of hGluN2B(P553L; and V558I) dysregulation

Crystal structures of the GluN1/GluN2B receptor show that the LBD, the pre-M1 helix, and the M1 helix are structurally coupled, indicating a role in receptor gating (Lee et al., 2014). Conserved residues GluN2B(P553; and V558) are part of the pre-M1 and M1 helix, respectively (Sobolevsky et al., 2009; Karakas and Furukawa, 2014; Figure 1A), and despite their physical proximity, the mutation hGluN2B(P553L) rendered the transfected cells virtually glutamate nonresponsive while hGluN2B(V558I) receptors exhibited similar current responses to 1 mM glutamate to the WT, although with a prominent desensitization and a decreased *P*_*o*_ (Supplementary Figure S2, Figures 4D,E). Surface expression of YFP-hGluN1/hGluN2B(P553L) receptors was not altered (Figure 2E; however, see Ogden et al., 2017), who show a moderate reduction (by 33%) indicating that minimal current responses to 1 mM glutamate in hGluN1/hGluN2B(P553L) receptors are not due to the lack of surface expression but rather a functional alteration in the receptor. We may hypothesize that the loss of function in hGluN1/hGluN2B(P553L) receptors may be attributed to receptor desensitization since rat GluN1/GluN2A(P552R) receptors, homologous in position to GluN2B(P553), desensitize profoundly (by 98.5%). It is less likely that it is due to altered agonist potency and *P*_*o*_ since rat GluN1/GluN2A(P552R) and rat GluN1/GluN2B(P553R) receptors have the EC_50_ value for both glutamate and glycine decreased in combination with an increased *P*_*o*_ (Ogden et al., 2017). Augmented desensitization of hGluN1/hGluN2B(V558I) receptors agrees well with results of previous experiments with chimeric receptors containing desensitizing GluN2A and non-desensitizing GluN2C subunit, as well as single-point mutations, which have identified the pre-M1 and M1 region as critical for rapid glycine- and Ca^2+^-independent desensitization of NMDARs (Krupp et al., 1998; Villarroel et al., 1998; Thomas et al., 2006). In addition, this region has been shown to influence *P*_*o*_ of the NMDAR channels (Ogden et al., 2017).

**Figure 7:**
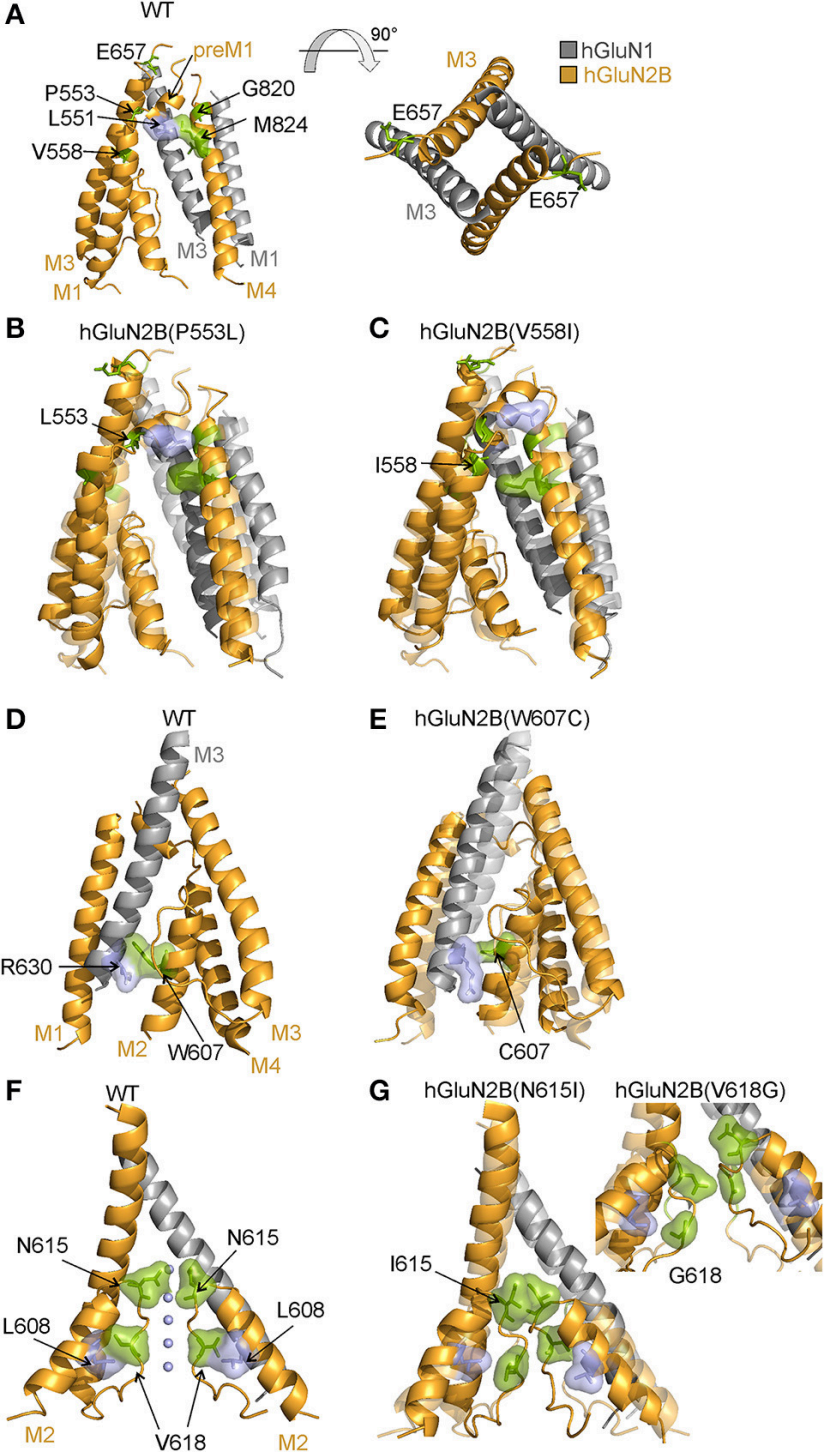
Structural consequences of hGluN2B(P553L; V558I; W607C; N615I; and V618G) mutations. **(A)** Cartoon representation of the homology model of the membrane domain of the WT receptor built from the rat GluN1/GluN2B crystallographic data (Karakas and Furukawa, 2014; Lee et al., 2014) (GluN1 in gray; GluN2B in orange). MD simulation (of the WT receptor) indicates a van der Waals contact between hGluN2B(L551; and M824) that is important for the arrangement of the M1, pre-M1, and M3 helices. On the right, the membrane domains (M3 helices) of the NMDAR channel are viewed from the extracellular side. **(B)** Shows results of MD simulation of the hGluN1/hGluN2B(P553L) (deep colors) and WT receptor (light colors). Comparison of both superimposed models indicates that in hGluN1/hGluN2B(P553L) receptors the helical structure of the pre-M1 is prolonged and as a result contact between hGluN2B(L551; and M824) is disrupted and M1 and M4 helices repositioned. **(C)** Shows results of MD simulation of the hGluN1/hGluN2B(V558I) (deep colors) and WT receptor (light colors). The models indicate that in hGluN1/hGluN2B(V558I) receptors the helical structure of the M1 is prolonged and as a result contact between hGluN2B(L551; and M824) is disrupted and M1 and M4 helices repositioned. **(D,E)** Models of the WT and hGluN1/hGluN2B(W607C) receptors as deduced from the MD simulation. In the WT a van der Waals contact between hGluN2B(R630; and W607) is replaced by a weak contact between hGluN2B(R630) and smaller cysteine in hGluN2B(W607C). This change results in altered position of the M3 and M2 helices of the hGluN2B subunits. **(F,G)** Shows models of the WT, hGluN1/hGluN2B(N615I; and V618G) channel permeation pathway. The position of ions in the channel of the WT receptor was built from the crystallographic data of the structurally related K+ channel, pdb id 1k4c (Zhou et al., 2001). MD simulation of the WT indicates importance of a van der Waals contact between hGluN2B(L608) and hGluN2B(V618) that allow optimal orientation and precise positioning of the hGluN2B M2 loop in the channel permeation pathway. Permeant ions (Na^+^/K^+^/Ca^2+^; indicated by blue spheres) interact with polar side chains of hGluN2B(N615) residues and backbone carbonyl groups of hGluN2B(V618). Simulation of the hGluN1/hGluN2B(N615I) receptor indicates that bulky nonpolar isoleucine residue lost the ability to coordinate the ions and occlude the permeation pathway as a result of van der Waals interaction. In addition contact of hGluN2B(L608) and hGluN2B(V618) is disrupted and as a consequence the M2 loop is destabilized with frequent transitions in between diverse conformations of the backbone. Similar destabilization effect is seen in hGluN1/hGluN2B(V618G) receptors.

We have employed computational methods to infer the structural consequences of *de novo* missense mutations based on the liganded state with the channel just prior to opening (Vyklicky et al., 2015) as deduced from the available crystal structures of the heterotetrameric GluN1/GluN2B receptor (Furukawa et al., 2005; Karakas and Furukawa, 2014; Lee et al., 2014). In the pre-open liganded state, the pre-M1 helix controls the spacing between M1 and M4 helices. It is also involved in further transmitting the mechanical signal from ligand-induced LBD reorientation through the pore-forming M3 helix to the M1 and M4 helices (Figure 7A). Specifically, the hGluN2B(P553) residue interacts weakly with L650 and F653 of the M3 helix. MD simulation further suggests that the hGluN2B(P553L) mutation leads to a formation of a nearly continuous M1 helix as a result of removing the helix-terminating proline residue (Figure 7B). The hGluN2B(V558) is mediating an interaction with surrounding hydrophobic residues of M3 helix in the liganded state. The MD simulation suggests that the hGluN2B(V558I) mutation induces stronger interaction of these residues, leading to reorientation of the pre-M1 helix from its horizontal position (Figure 7C). As a result, the M1/M4 interaction is weakened.

### Mechanisms of hGluN2B(W607C; N615I; and V618G) dysregulation

Cysteine accessibility to sulfhydryl reagents indicates that conserved residues hGluN2B(W607; N615; and V618) are exposed to the lumen of the NMDAR ion channel (Kuner et al., 1996). Our results show that mutations of these residues underlie multiple effects: hGluN1/hGluN2B(W607C) receptors have a normal or moderately decreased surface expression, a decreased agonist potency, and a decreased *P*_*o*_; whereas hGluN1/hGluN2B(V618G) have only a decreased *P*_*o*_ (Figures 2–4). Similarly to hGluN1/hGluN2B(W607C; and V618G) receptors, hGluN1/hGluN2B(N615I) receptors exhibit reduced responses to 1 mM glutamate (by 72%, see Supplementary Figure S2), although without an obvious change in trafficking and/or the functional parameters studied.

A characteristic feature of the hGluN1/hGluN2B(N615I) receptors was an accelerated rate of recovery from MK-801 inhibition (see Supplementary Figure S1), indicating structural differences in the ion channel pathway between WT and mutated receptors. There is compelling evidence that asparagine residues [GluN1(N616), GluN2A(N614), GluN2B(N615)] and adjacent residues including tryptophan [GluN2A(W606) or GluN2B(W607)] control permeability and block by Mg^2+^ (Williams et al., 1998) and, in addition, GluN2B(N615) controls single-channel conductance (for review see Dingledine et al., 1999). We therefore performed additional experiments to analyze Mg^2+^ block and single-channel currents. In agreement with recent data of Mullier et al. (2017), our analysis showed that Mg^2+^ sensitivity was decreased in hGluN1/hGluN2B(N615I; and W607C) receptors. Since the structural determinants of external Mg^2+^ block are similar to those governing Ca^2+^ permeability through NMDA receptor channels, it is possible that hGluN1/hGluN2B(W607C; N615I; and V618G) receptors may also have altered Ca^2+^ permeability. However, this was not possible to assess reliably due to very small current responses (Supplementary Figure S2). Similarly to Mg^2+^ block, the mean amplitude of the single-channel current was decreased for hGluN1/hGluN2B(W607C; N615I; and V618G) receptor channels.

To infer structural changes induced by mutations in the M2 segment and re-entrant loop, we used our previous model of the TMD with the vestibule open (Vyklicky et al., 2015). The ion filter as well as the lower part of M1, M3, and M4 helices are not changed significantly between the model liganded pre-open and the open state. The used model of the WT NMDAR channel is compatible with previous estimates of the minimum cross-section of the channel permeation pathway in the conducting states: 6.0 Å (Vyklicky et al., 1988), 5.5 Å (Villarroel et al., 1995), and ~4.5 × 5.7 Å (Zarei and Dani, 1995), and within the limits proposed for the channel diameter at the level of the activated extracellular vestibule, which was estimated to be 7.3 Å (Villarroel et al., 1995) and 11 Å (Sobolevskii and Khodorov, 2002). The W607 residue is involved in a stacking interaction with hGluN1(R630) residue in the M1 helix, which contributes to a proper arrangement of the M2 helices with respect to their surroundings (Figure 7D). MD of mutated model structures indicates that the W607C mutation interferes with this interaction and the relative orientation of the M2 helix (Figure 7E). The V618 residue interacting with the L608 contributes to the correct orientation of backbone carbonyl groups within the ion filter (Figure 7F). Structural effects of V618G mutation suggest a loss of this orientation, possibly resulting in lower selectivity and efficiency of ion transport (Figure 7G). The polar side chain of the N615 residue, homologous to Q586 of AMPAR, serves as the first layer coordinating the ion to be transported. The hGluN2B(N615I) mutation will lead to a change in channel selectivity and ion permeation through the channel (Figure 7G).

### Mechanisms of hGluN2B(S628F) dysregulation

Reduced responses to 1 mM glutamate in HEK293T cells transfected with hGluN1/hGluN2B(S628F) receptors (Supplementary Figure S2) correlate with their reduced surface expression (Figure 2E, Supplementary Figure S3). hGluN2B(S628) is located at the intracellular segment of the M3 helix and is conserved within the GluN2 family (Figure 1). We have shown earlier that amino acid residues within the M3 helix of GluN2B subunit (W635; S645; Y646; and T647) contribute to the regulation of the surface expression of NMDARs and alter the functional properties of NMDARs (Kaniakova et al., 2012). The quality-control at intracellular points is only poorly understood, and it is a matter of speculation whether the defect in surface expression described here for hGluN1/hGluN2B(S628F) receptors involves a mechanism similar to that of other amino acid mutations in the M3 helix or other TMD regions.

**Figure 8:**
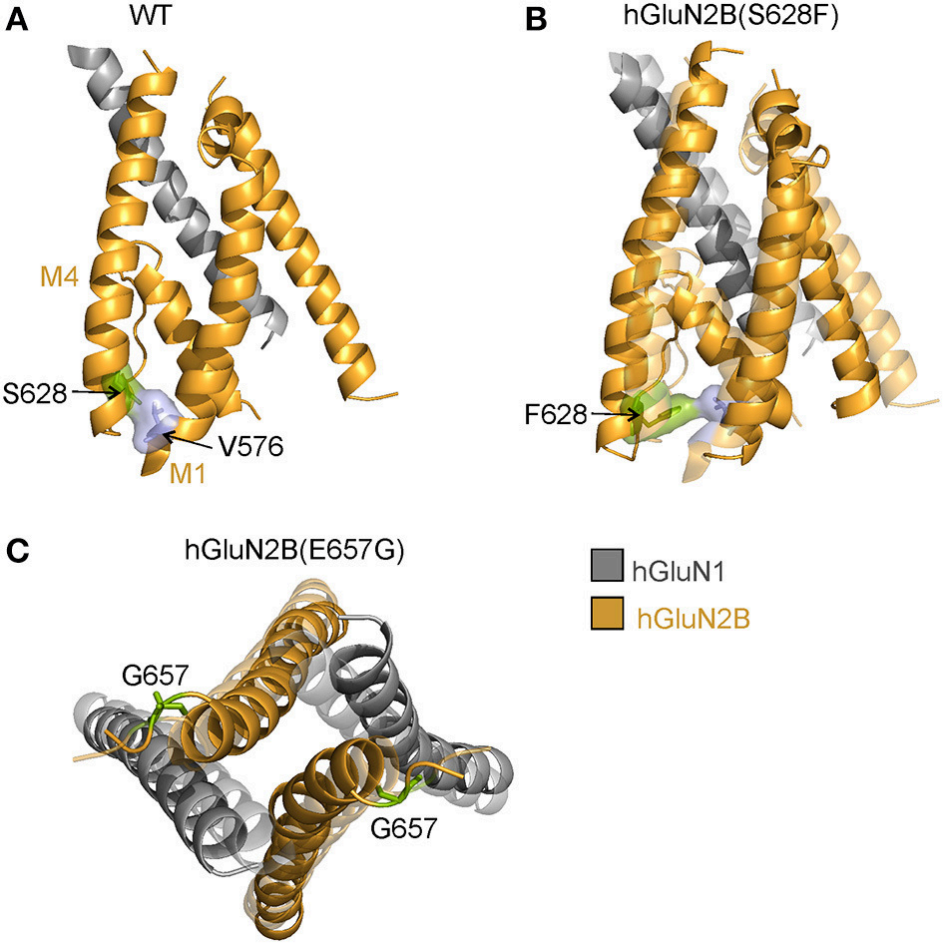
Structural consequences of hGluN2B(S628F; and E657G) mutations. **(A)** Cartoon representation of the homology model of the membrane domain of the WT receptor built from the rat GluN1/GluN2B crystallographic data (Karakas and Furukawa, 2014; Lee et al., 2014) (GluN1 in gray; GluN2B in orange). MD simulation of the WT receptor indicates a series of inter-helical zipper-like contacts spanning along the M1 and M4 helices. The contact between hGluN2B(S628) and hGluN2B(V576) is highlighted. **(B)** According to the MD simulation the hGluN2B(S628F) substitution leads to larger separation of M1 and M4 helices. Contrary to an expected stronger van der Waals interaction with the hGluN2B(V576) across helices the steric effect of larger hydrophobic phenylalanine residue leads to rearrangement of residues along the M4 helix and formation of a chain of van der Waals stabilized M4 residues. **(C)** The MD simulation of hGluN2B(E657G) mutation suggests closing of the ion channel due to a loss of specific interactions along the TMD-LBD hGluN2B linker.

Our MD simulation suggests that in the hGluN1/hGluN2B(S628F) receptors, the mutation of a polar and small serine for a larger nonpolar phenylalanine leads to steric hindrance, with the bottom part of the M1 helix altering the quaternary structure of the TMD. This may be the reason why they are offloaded from their trafficking to the cell surface (Figures 8A,B).

### Mechanisms of hGluN2B(E657G) dysregulation

Reduced responses to 1 mM glutamate in HEK293T cells transfected with hGluN1/hGluN2B(E657G) receptors with normal surface expression imply that the mutation introduced a severe functional defect (Supplementary Figure S2, Figure 2E). Glutamate residue hGluN2B(E657) is conserved within the GluN2, GluA, and GluK family and is part of the linker that connects the LBD and M3 helices. Rearrangement of M3 helices in the activated conformation of the receptor makes the central cavity of the channel accessible to ions, therefore implying a crucial role of the M3-S2 linkers in channel opening (Sobolevsky et al., 2004). Mutations in this region lead to profound alterations of the receptor channel function characterized by diminished *P*_*o*_ and altered transduction of agonist activation to channel opening (Kazi et al., 2014). Missing experimental electron density data in available crystal structures (Karakas and Furukawa, 2014; Lee et al., 2014) does not allow the localization of residues in the linker regions and related structural information is mostly missing, we therefore decided to explore linker structures computationally. Our data indicate that the overall effect of glycine substitution hGluN2B(E657G) on NMDAR gating can be explained by an interaction of specific residues at a site located in the proximity of the M3 helices (Figures 7A, 8C).

### Mechanisms of hGluN2B(G820E; G820A; M824R; and L825V) dysregulation

Reduced responses to 1 mM glutamate observed in HEK293T cells transfected with genes encoding for hGluN1 and hGluN2B(G820A) subunits and a complete loss of responses in hGluN1/hGluN2B(G820E; and M824R) receptors are not due to altered surface expression since this was normal or even increased (Figure 2E). Small currents in hGluN1/hGluN2B(G820A) receptors precluded detailed functional examination. In contrast, responses to 1 mM glutamate in cells transfected with genes encoding for hGluN1 and hGluN2B(L825V) subunits were not significantly different from those observed in the WT although they had reduced *P*_*o*_ by 86% compared to the WT receptors.

**Figure 9:**
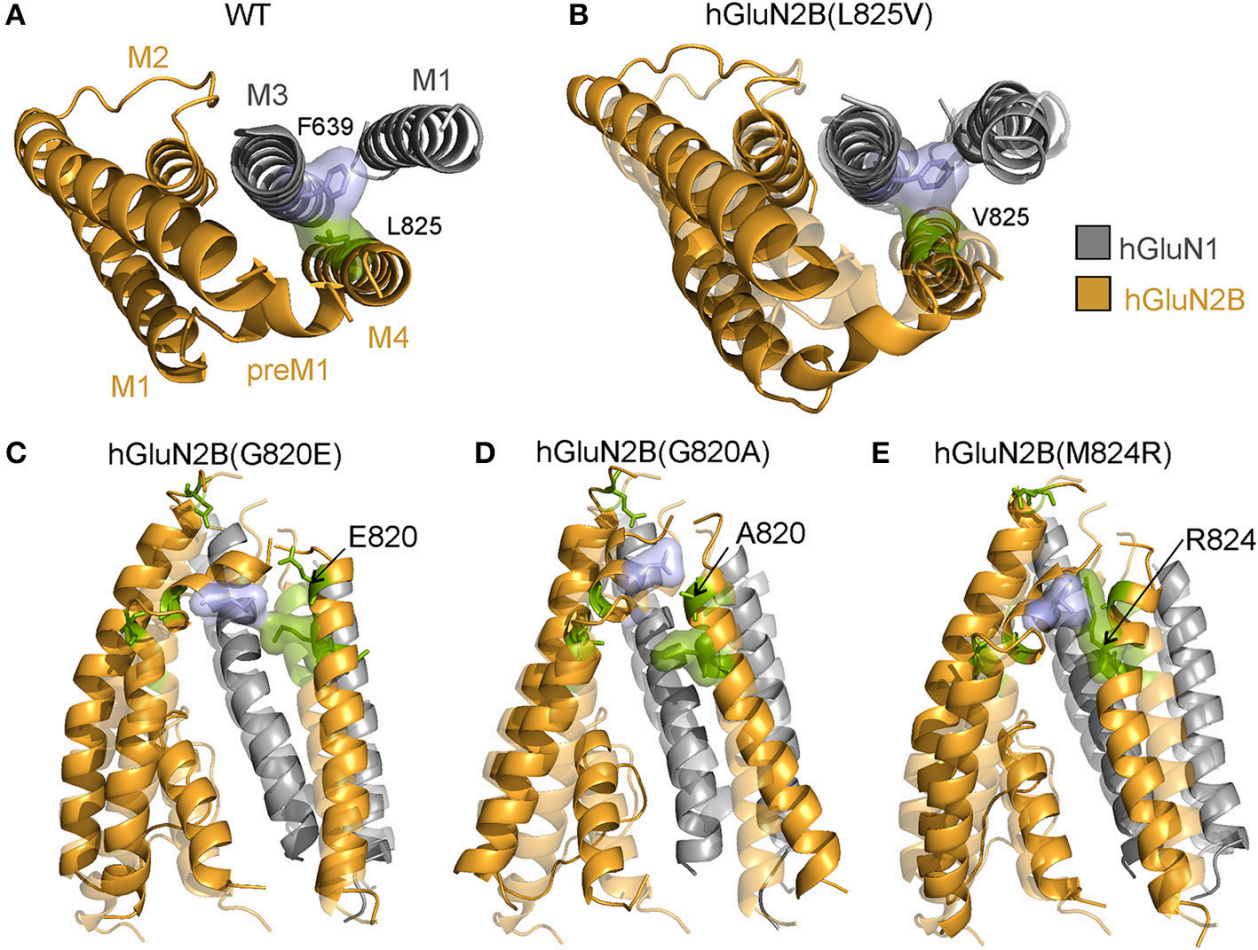
Structural consequences of hGluN2B(L825V; G820E; G820A; and M824R) mutations. **(A)** Cartoon representation of the homology model of the membrane domain of the WT receptor built from the rat GluN1/GluN2B crystallographic data (Karakas and Furukawa, 2014; Lee et al., 2014) (GluN1 in gray; GluN2B in orange). MD simulation of the WT receptor identified a van der Waals contact between hGluN1(F639) and hGluN2B(L825) involved in interaction of hGluN1 M3 helix with the hGluN2B M4 helix**. (B)** The hGluN2B(L825V) substitution for a smaller aliphatic residue leads to weaker van der Waals interaction with the hGluN1(F639) resulting in a slight separation of the interacting helices. However, the structural effects are inconclusive. **(C,D)** MD revealed that hGluN2B(G820) serves as the last residue in the M4 helix thus allowing the necessary extension of the preceding linker in response to the LBD reorientation. The hGluN2B(G820A; and G820E) mutations remain in the helical context and the introduced side chains interact with residues from the pre-M1 helix. This changes the pre-M1 horizontal position as well as relative positions of M1 and M4 helices. **(E)** The hGluN2B(M824) residue is involved in an interaction with hGluN2B(L551) of the pre-M1 helix in the liganded state. The hGluN2B(M824R) mutation prevents the pre-M1 interaction and leads to altered relative position of M1 and M4 helices and consequently to inefficient propagation of the mechanical signal from the LBD.

Structurally conserved residues hGluN2B(G820, M824, and L825) (Figure 1) are located in mutual proximity in the M4 helix. Several lines of evidence indicate that M1 and M4 helices are located at the periphery of the TMD and seem to have a modulatory role in channel opening and closing, which is primarily mediated by M3 helices that form the central cavity of the channel in the activated receptor (Villarroel et al., 1998; Sobolevsky et al., 2004; Ogden and Traynelis, 2013; Karakas and Furukawa, 2014). The hGluN2B(L825) residue interacts with the F639 from the M1 helix of hGluN1 in the liganded state (Figure 9A). The hGluN2B(L825V) mutation leads to a weaker interaction with the hGluN1(F639), although structural effects are inconclusive (Figure 9B). MD revealed that hGluN2B(G820) serves as the last residue in the M4 helix, thus allowing the necessary extension of the preceding linker in response to the LBD reorientation. The hGluN2B(G820E; and G820A) mutations remain in the helical context and their side chains interact with the pre-M1 helix, shifting it from its horizontal position and changing the relative positions of M1 and M4 helices (Figures 9C,D). In the liganded state, the hGluN2B(M824) residue is involved in an interaction with hGluN2B(L551) of the pre-M1 helix (Figure 7A). As a consequence of the hGluN2B(M824R) mutation, the pre-M1 interaction is not formed (Figure 9E); this leads to an altered relative position of M1 and M4 helices, which in turn results in inefficient propagation of the mechanical signal from the LBD.

### Pharmacology of mutated receptors

Therapeutic strategies for the development of new effective drugs for rectifying loss-of-function variants associated with diminution of the receptor *P*_*o*_ may encompass PE-S and its derivatives, since these compounds have a positive allosteric effect that is specifically mediated by the increase in the receptor *P*_*o*_ (Horak et al., 2004; Korinek et al., 2011). Both an endogenously occurring neurosteroid PE-S (Wu et al., 1991; Bowlby, 1993) and its more potent synthetic analog AND-hSuc (Krausova et al., 2018) were approximately as effective at WT as at the hGluN1/hGluN2B(V558I; W607C; V618G; and G820A) receptors. Interestingly, both steroids potentiated the amplitude of tonic and synaptic-like responses more profoundly when studied at hGluN1/hGluN2B(L825V) receptors, indicating their prospective use in personalized therapies. It is likely that the augmented potentiating effect for PE-S is not specifically associated with residues at the TMD, since similar increases have been recently shown for receptors mutated in the LBD of the GluN2A subunit (V685G; and D731N) (Swanger et al., 2016).

Functional defects described here in the NMDAR apply for cases in which both alleles in a patient were mutated at exactly the same site. This is expected to be rare, in contrast to heterozygous individuals, who are much more likely to carry only one mutated allele. In this case, the expected genotypic ratio predicts that each neuron will contain 25% of WT receptors, 50% of receptors composed of one hGluN2B subunit mutated and one non-mutated, and 25% of receptors comprised of both hGluN2B subunits mutated.

To build a conception of the degree of the NMDAR defect, we may use as an example the mutation-induced decrease in the receptor *P*_*o*_ (*P*_*o*_ = 10% for the WT and *P*_*o*_ = 1% for receptors with both hGluN2B subunits mutated). Based on the Mendelian laws of inheritance and the law of dominance, we may predict that the overall activity of NMDARs will be diminished, depending on whether the mutation is recessive, independent, or dominant, to 77.5, 55, and 32.5%, respectively, of the activity in the healthy subjects. These calculations indicate that for a full pharmacological rectification of the NMDAR defect, a potentiating effect of 22.5, 45, and 67.5% would be required. Since most neurons express more than one type of GluN2/GluN3 subunits (Monyer et al., 1994), the expected rectifying potentiation effect may be even smaller. However, further studies are required to understand how the degree of NMDAR hypofunction is linked to various clinical symptoms.

## Author contributions

VV and LV designed the experiments; VV, BKr, BKy, ML, TS, and MK performed electrophysiological experiments; BKr, SD, and MH performed trafficking experiments; VV, BKr, and LV analyzed the data; JC performed molecular modeling; HC and EK synthetized the steroids; LV wrote the paper.

### Conflict of interest statement

The authors declare that the research was conducted in the absence of any commercial or financial relationships that could be construed as a potential conflict of interest.
