# Supplementary material for: Surface Expression, Function, and Pharmacology of Disease-Associated Mutations in the Membrane Domain of the Human GluN2B Subunit
Source: Front Mol Neurosci. 2018 Apr 6;11:110. doi: 10.3389/fnmol.2018.00110 (PMC5897658; doi:10.3389/fnmol.2018.00110)
Supplement: Supplementary file 1 [file DataSheet1.docx]

Supplementary Material

**Surface expression, function, and pharmacology of disease­associated mutations in the membrane domain of the human GluN2B subunit**

**Running title:** Disease-associated mutations of NMDA receptors

**Author listing:**

Vojtech Vyklicky^1,4^, Barbora Krausova^1,4^, Jiri Cerny^1^, Marek Ladislav^1,3^, Tereza Smejkalova^1^, Bohdan Kysilov^1^, Miloslav Korinek^1^, Sarka Danacikova^1,3^, Martin Horak^1^, Hana Chodounska^2^, Eva Kudova^2^, Ladislav Vyklicky^1^

^1^ Institute of Physiology CAS, Videnska 1083, 142 20 Prague 4, Czech Republic

^2^ Institute of Organic Chemistry and Biochemistry CAS, Flemingovo nam. 2, 166 10 Prague 6, Czech Republic

^3^ Faculty of Science, Charles University, Albertov 6, 128 43 Prague 2, Czech Republic

^4^ Co-first authors

**Correspondence:** Ladislav Vyklicky: ladislav.vyklicky@fgu.cas.cz


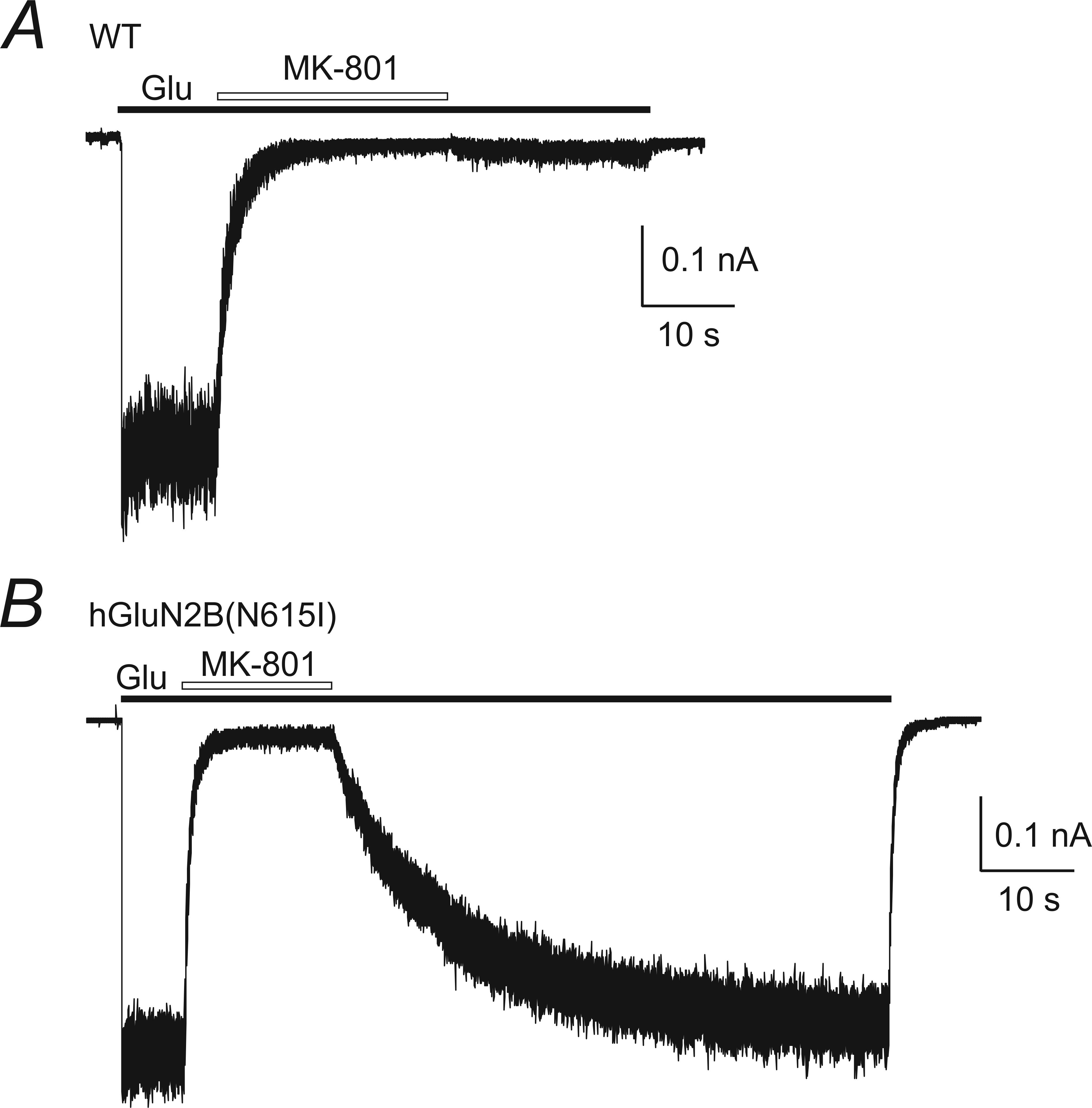


## Supplementary Figure S1

Differences in the rate of recovery from MK-801 block. Representative recordings from WT and hGluN1/hGluN2B(N615I) receptors expressed in HEK293T cells. Responses were induced by fast application of 1 mM glutamate in the continuous presence of 30 µM glycine and inhibited by 1 µM MK-801. In the WT, the responses made following MK-801 application did not appreciably recover during 20 s of glutamate application – indicating virtually irreversible MK-801 block **(A)**. In contrast, hGluN1/hGluN2B(N615I) responses recovered completely with τ = 12.9 s **(B)**. Holding potential in both cases was -60 mV.


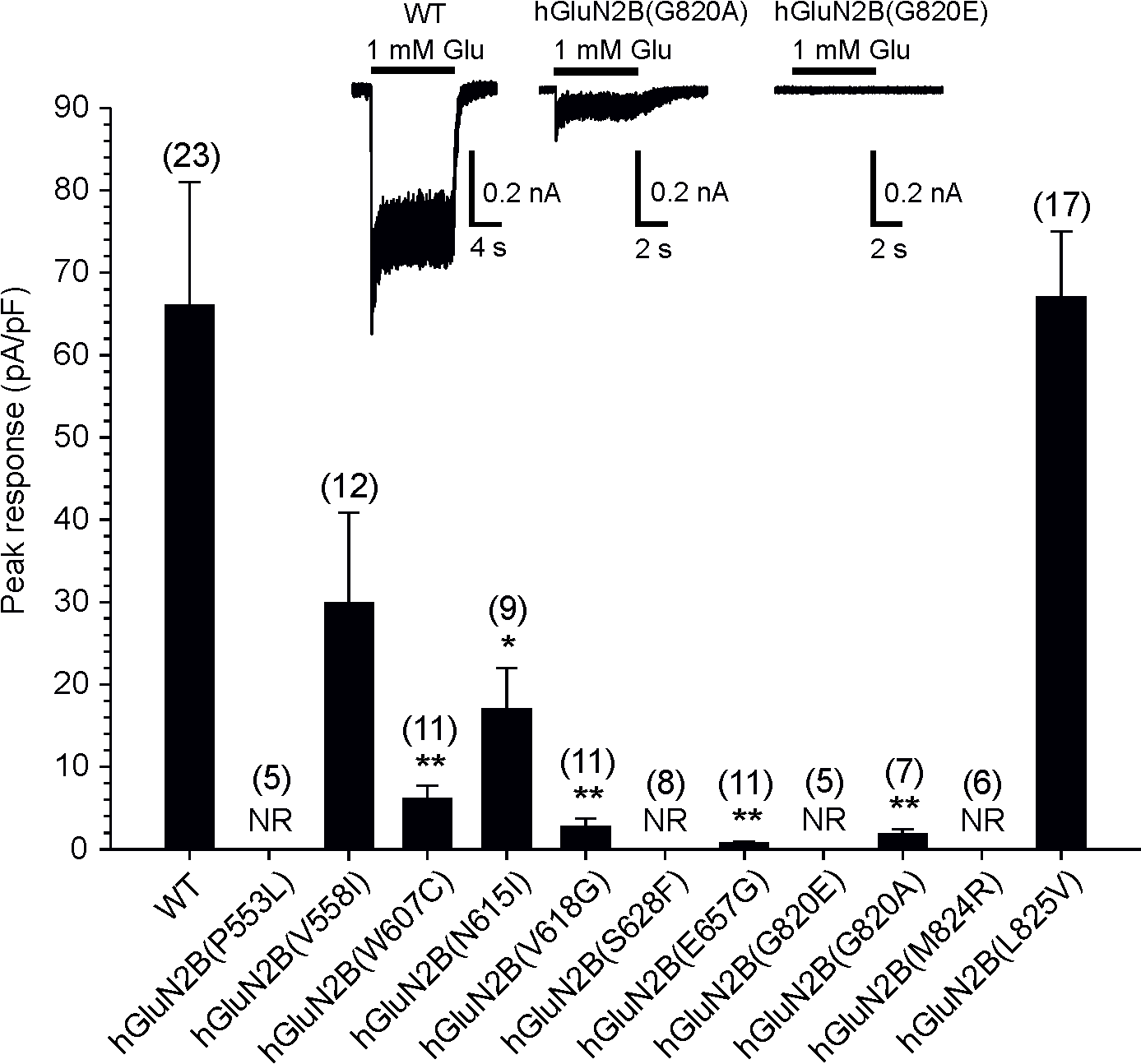


## Supplementary Figure S2

Mutations in the TMD of hGluN2B subunit affect the amplitude of responses to 1 mM glutamate. Representative whole-cell recordings of currents induced in HEK293T cells expressing WT, hGluN1/hGluN2B(G820A; and G820E) receptors. Glutamate (1 mM, black bar) was applied for 10 s. Cells were bathed in glycine (30 µM). Graph shows mean current amplitudes ± SEM (*n*) evoked by 1 mM glutamate and normalized with respect to HEK293T cell capacitance. Mutations that did not show detectable glutamate-activated currents are indicated NR (non-responding). Differences in mean values among the treatment groups were statistically significant - Kruskal-Wallis one-way ANOVA on Ranks (*p*< 0.001). Mann-Whitney Rank Sum Test was used to assess significance level compared to the WT; * *p* = 0.001-0.05; ** *p* < 0.001.


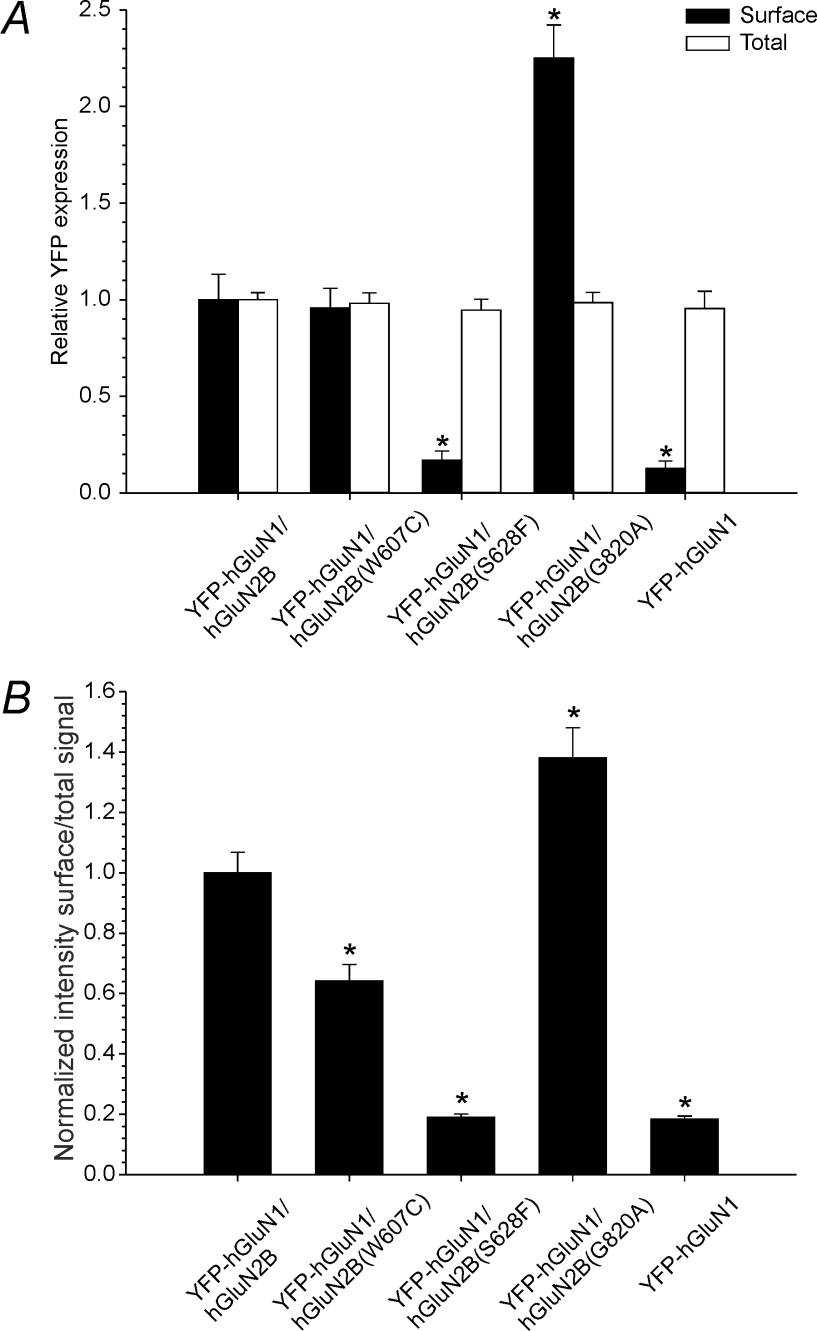


## Supplementary Figure S3

Mutations in the TMD of hGluN2B subunit affect NMDAR surface expression. **(A)** A quantitative assay of surface and total expression was used to determine relative NMDAR expression in COS-7 cells transfected with only YFP-hGluN1 subunit for control (YFP-hGluN1) or both the YFP-hGluN1 and hGluN2B (WT) or mutated hGluN2B subunit. Data show mean ± SEM from three independent experiments. * *p* < 0.05 relative to WT, one-way ANOVA (Multiple comparisons *versus* WT - Dunnett's method). **(B)** Immunofluorescence microscopy was used to determine relative surface-to-total NMDAR levels in HEK293T cells transfected with only YFP-hGluN1 subunit for control (YFP-hGluN1) or both the YFP-hGluN1 and hGluN2B (WT) or mutated hGluN2B subunit. Data show mean ± SEM; *n* = 35 in two experiments. * *p* < 0.05 relative to WT, one-way ANOVA (Multiple comparisons *versus* WT - Dunnett's method).


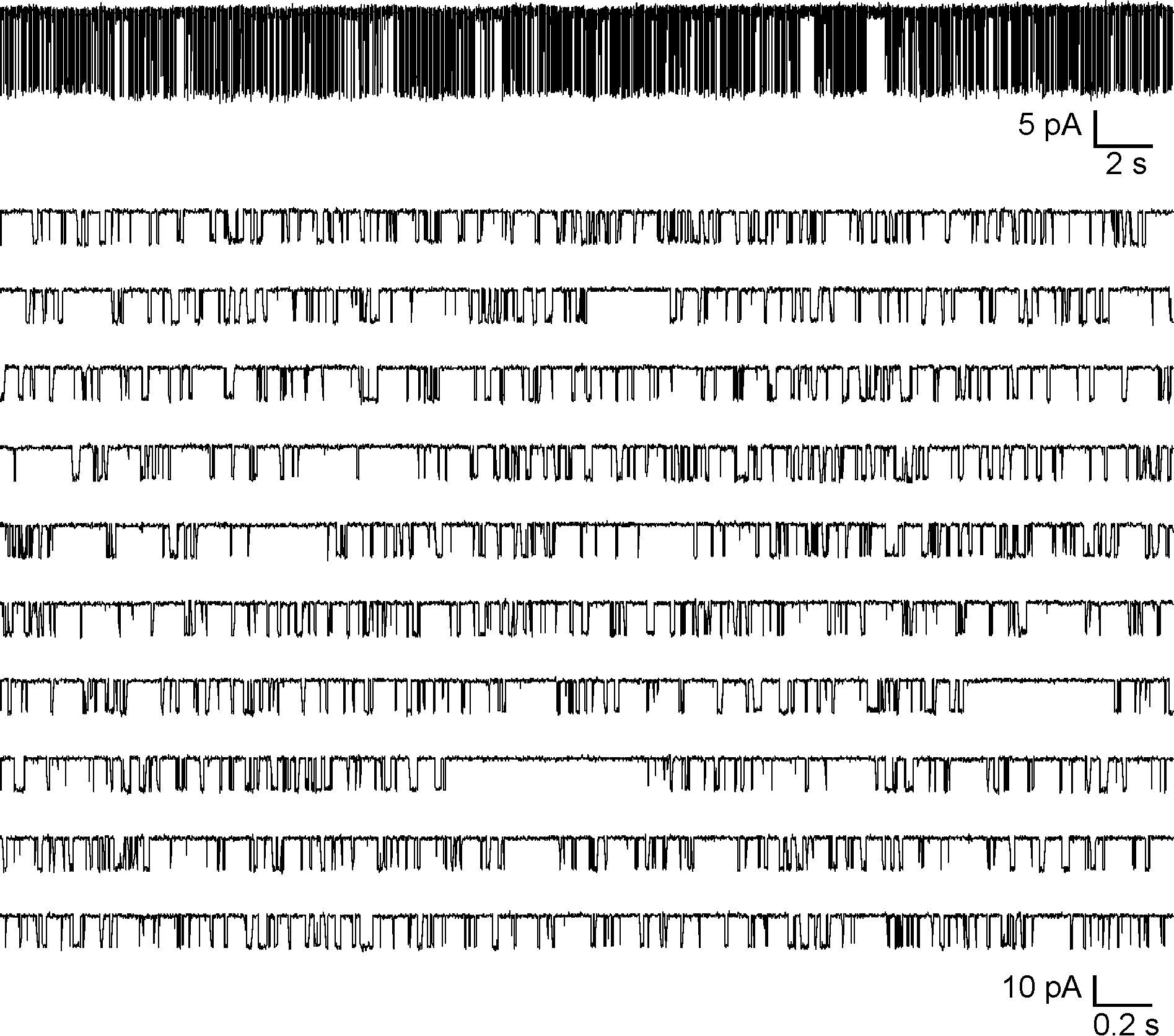


## Supplementary Figure S4

Representative steady-state cell-attached patch recordings from HEK293 cells expressing human WT receptors (hGluN1/hGluN2B). Unitary currents were activated in these patches by 1 mM glutamate and 0.1 mM glycine.

**Methods**

Steady-state single-channel recordings were done at the room temperature using the cell-attached patch-clamp technique. Recordings were analog-filtered at 10 kHz with a 4-pole Bessel filter and sampled at 25 kHz. Bath solution was identical to control solution from whole-cell recordings. Thick-wall borosilicate patch pipettes were pulled and fire-polished achieving resistances between 10 and 20 MΩ and filled with extracellular solution containing (in mM) 160 NaCl, 2.5 KCl, 1 mM EDTA and 10 HEPES (pH-adjusted to 7.3 with NaOH) as well as 0.1 glycine and 1 glutamate. Recordings were performed in the absence of divalent ions (1 mM EDTA) to achieve unitary current**.** Inward openings were detected by applying a pipette potential of +100 mV (estimated holding potential –130 mV).

QuB software (University at Buffalo, NY, USA) was used for the analysis of single-channel cell-attached records. To be sure that we analyze only patches with a single active channel we chose records that were sufficiently long (>5000 events) and entirely free of overlapping openings (99% confidence) (Colquhoun and Hawkes, 1990). The lowest Po was 0.02 which means that simultaneous opening of two channels should be visible approx. every 220 events, which is over 20-fold fewer events than our chosen minimum. The data were idealized by QuB software with an SKM algorithm (digital low-pass filter at 12.5 kHz, 0.12 ms dead time). Analysis of idealized data was performed with the maximum interval likelihood (MIL) algorithm (Qin et al., 1997; Amico-Ruvio and Popescu, 2010).

**Results**

From twelve different cell-attached patches containing only one active hGluN1/hGluN2B channel, we estimated the probability of opening of 10.6 ± 2.9%. Mean open time was 4.5 ± 0.6 ms and mean closed time 73.9 ± 15.1 ms.

**References**

Amico-Ruvio, S.A., and Popescu, G.K. (2010). Stationary gating of GluN1/GluN2B receptors in intact membrane patches. *Biophys J* 98(7)**,** 1160-1169. doi: 10.1016/j.bpj.2009.12.4276.

Colquhoun, D., and Hawkes, A.G. (1990). Stochastic properties of ion channel openings and bursts in a membrane patch that contains two channels: evidence concerning the number of channels present when a record containing only single openings is observed. *Proc R Soc Lond B Biol Sci* 240(1299)**,** 453-477.

Qin, F., Auerbach, A., and Sachs, F. (1997). Maximum likelihood estimation of aggregated Markov processes. *Proc Biol Sci* 264(1380)**,** 375-383. doi: 10.1098/rspb.1997.0054.
