# Supplementary figures and images for: Surface Expression, Function, and Pharmacology of Disease-Associated Mutations in the Membrane Domain of the Human GluN2B Subunit
Source: Front Mol Neurosci. 2018 Apr 6;11:110. doi: 10.3389/fnmol.2018.00110 (PMC5897658; doi:10.3389/fnmol.2018.00110)

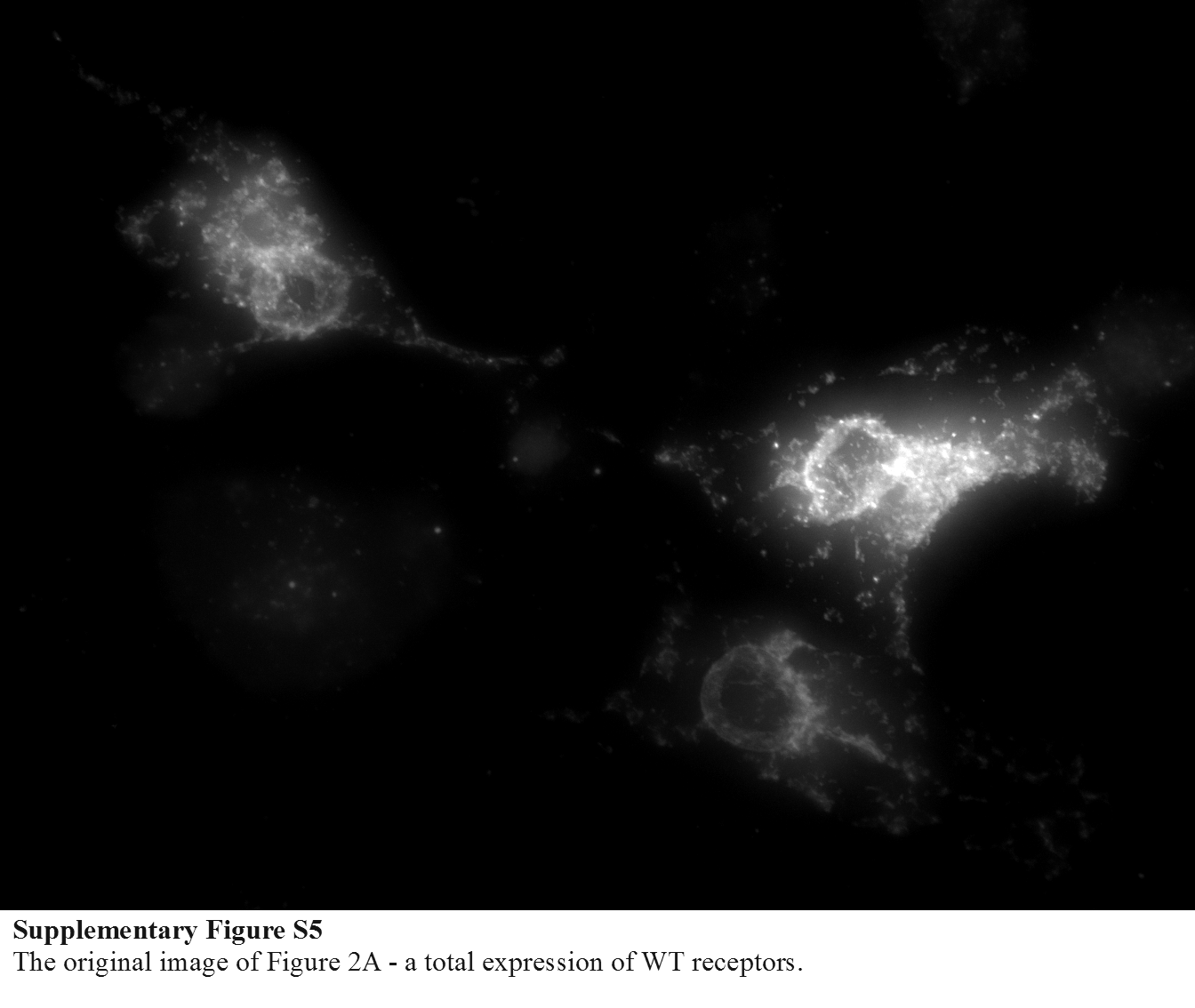

Supplement: Supplementary file 2 [file Image5.tif]

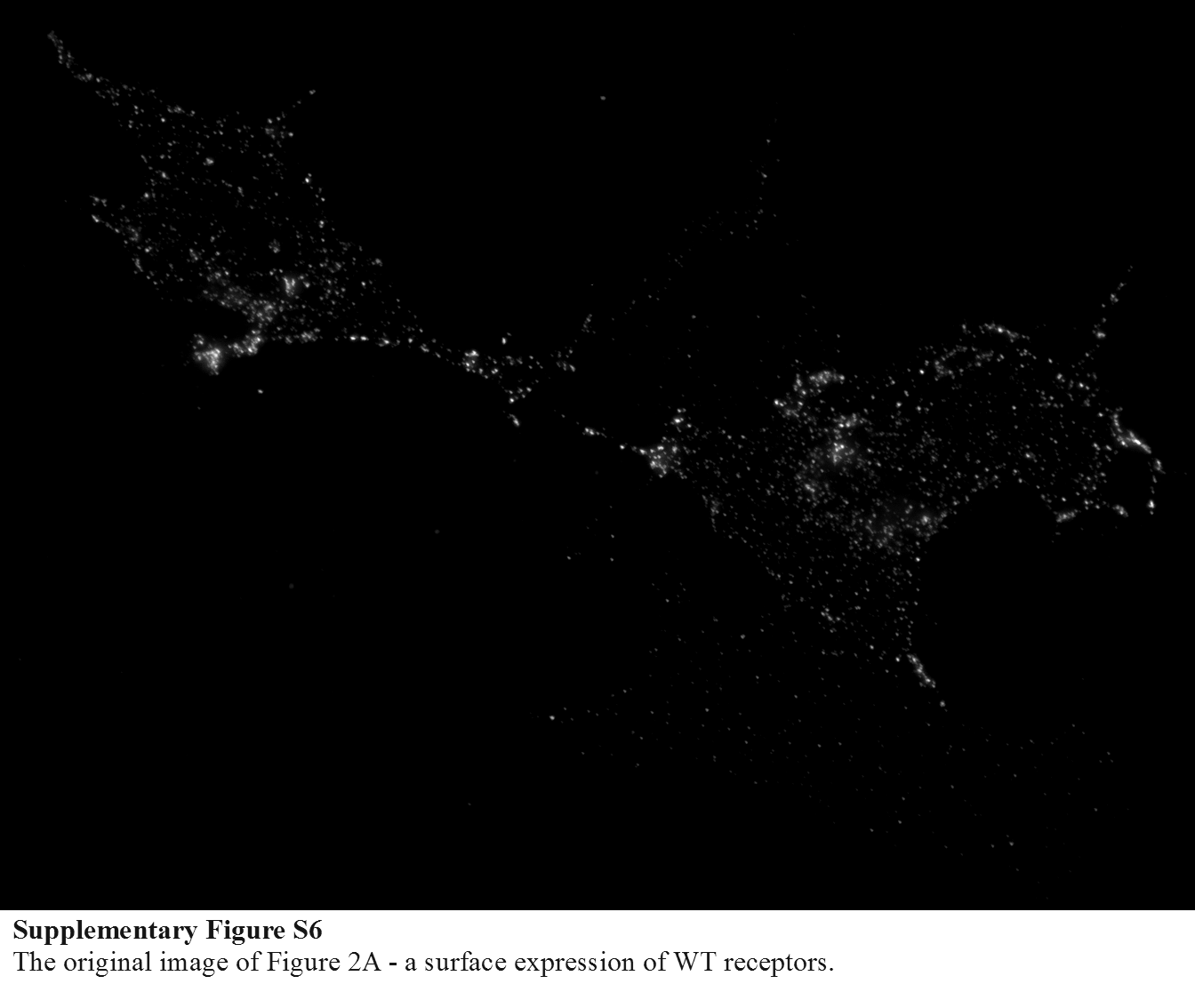

Supplement: Supplementary file 3 [file Image6.tif]

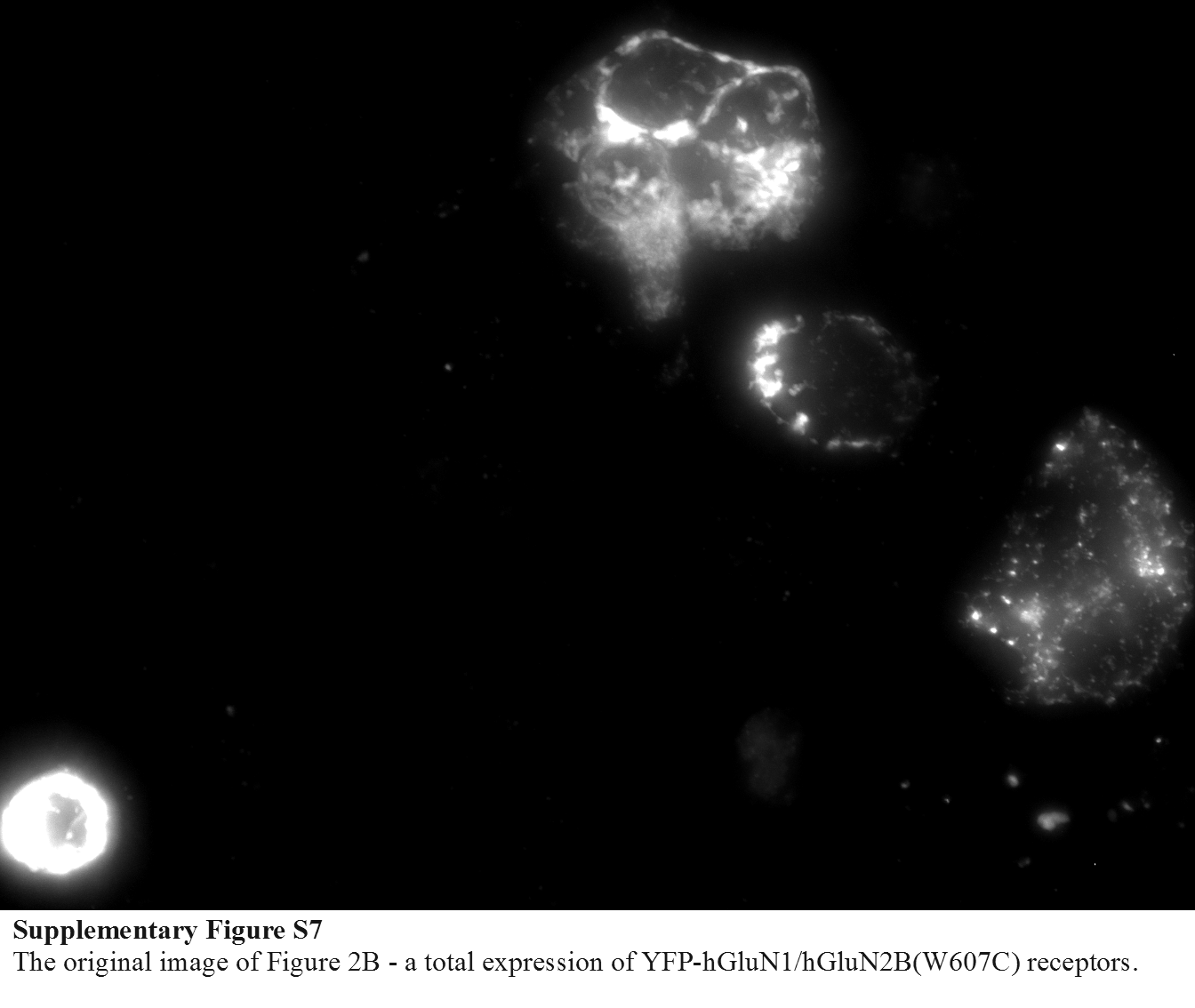

Supplement: Supplementary file 4 [file Image7.tif]

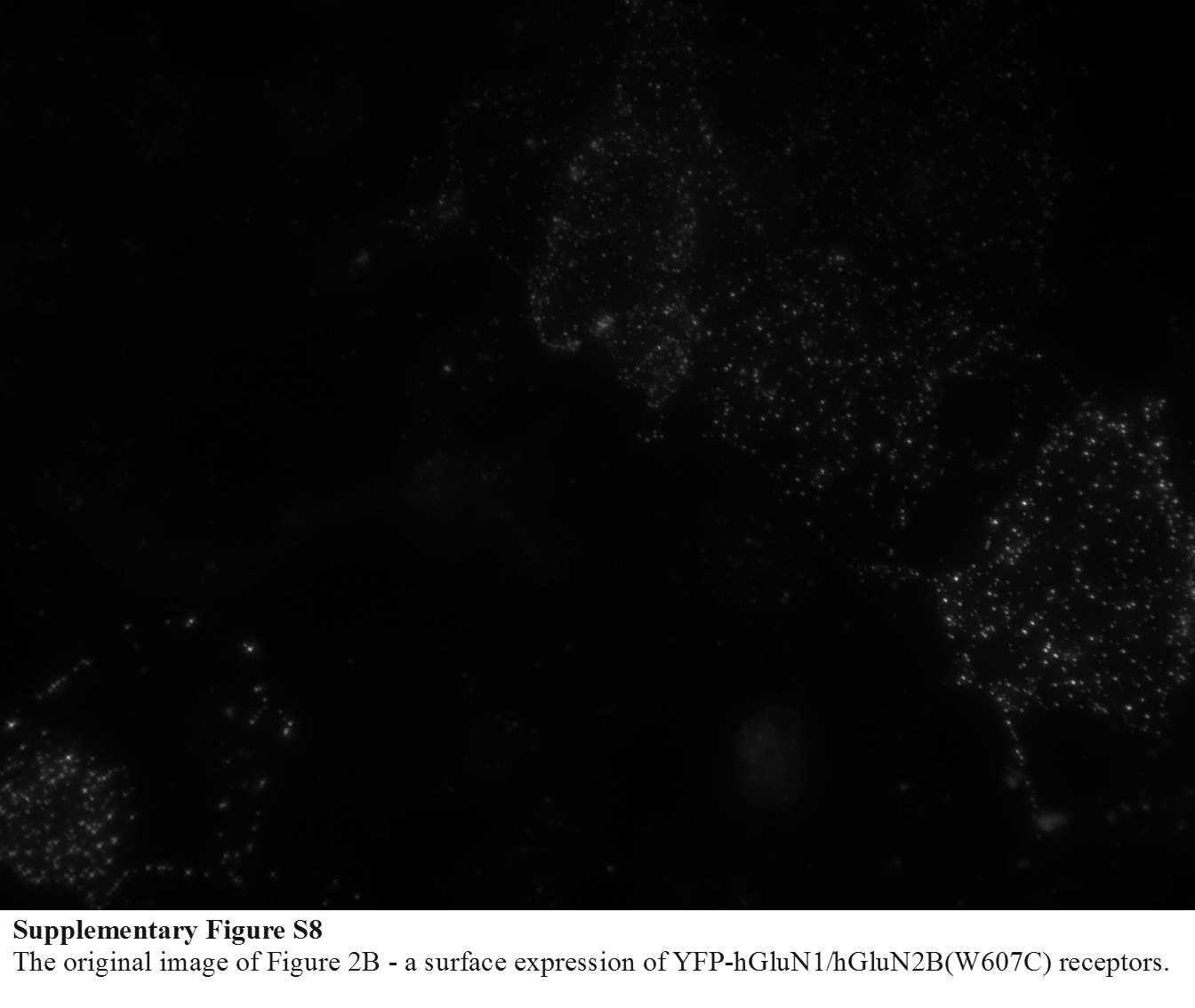

Supplement: Supplementary file 5 [file Image8.tif]

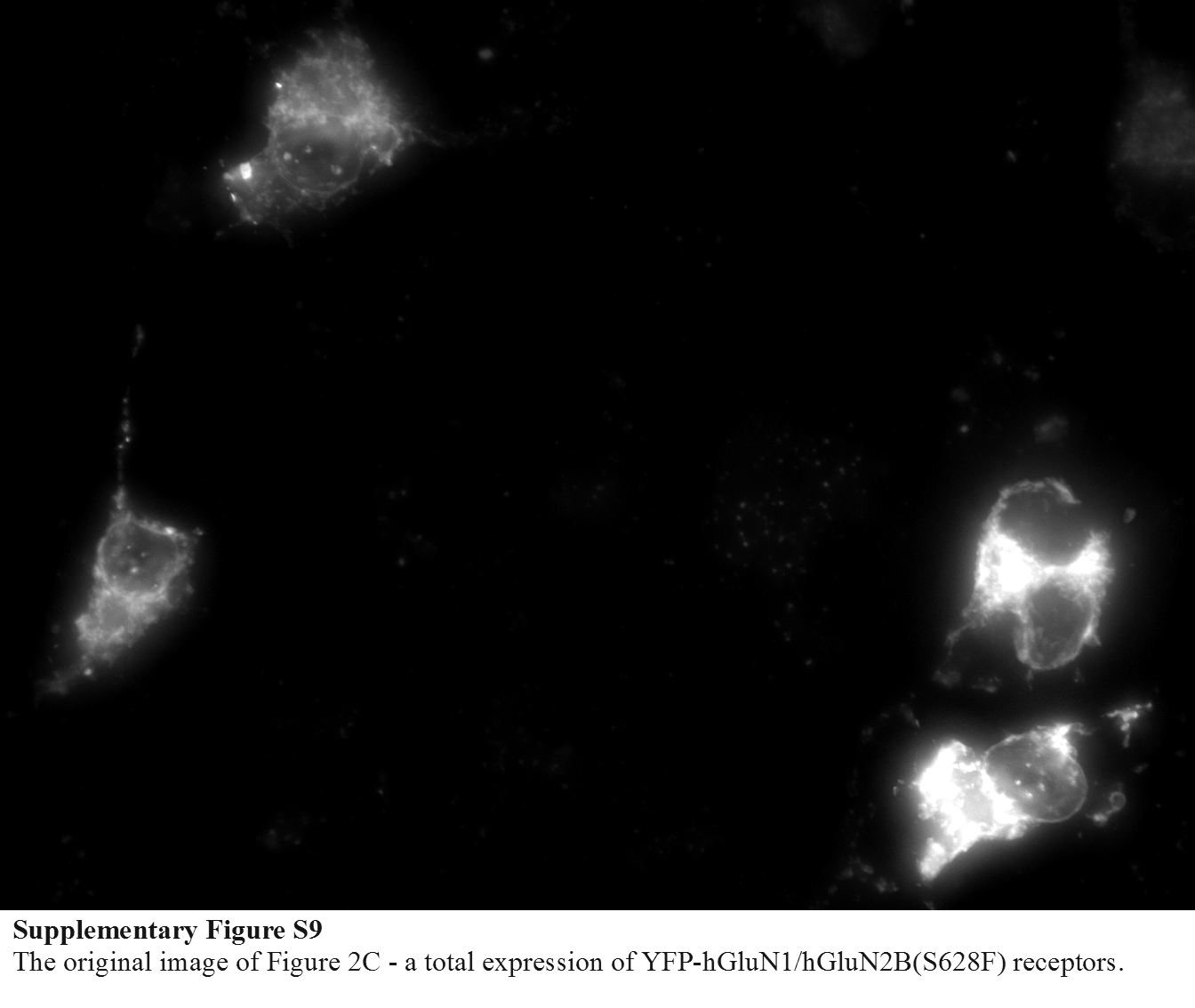

Supplement: Supplementary file 6 [file Image9.tif]

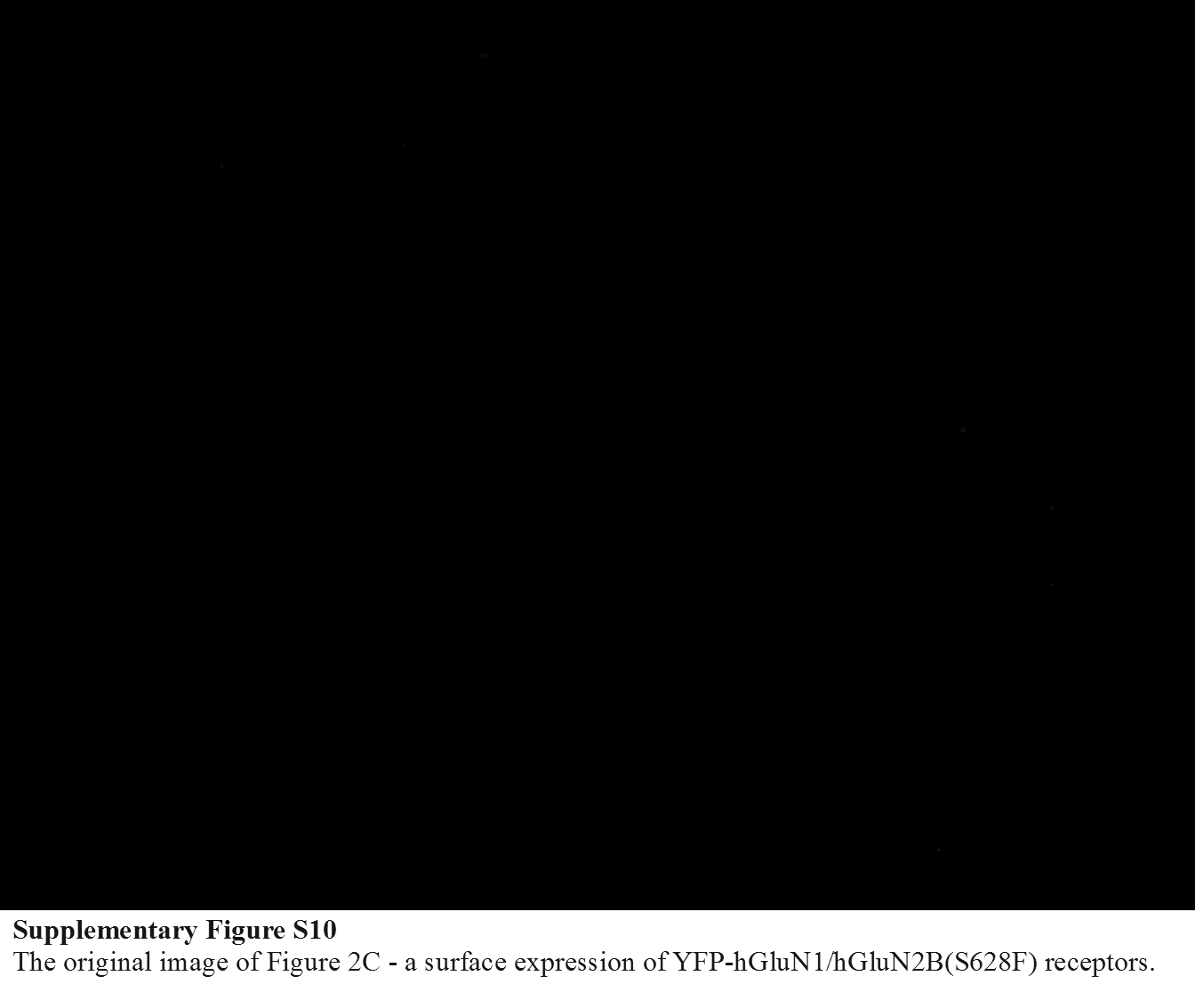

Supplement: Supplementary file 7 [file Image10.tif]

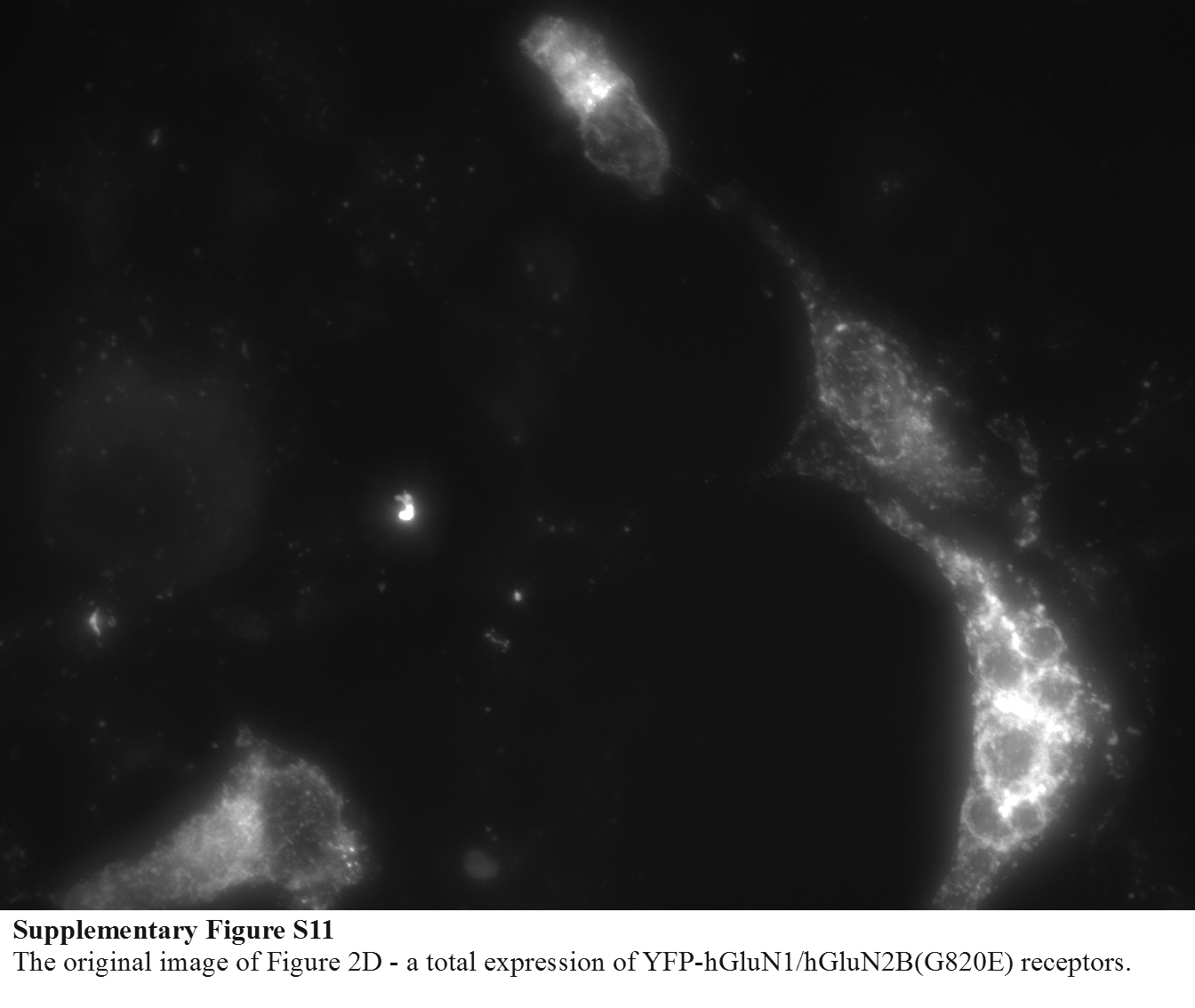

Supplement: Supplementary file 8 [file Image11.tif]

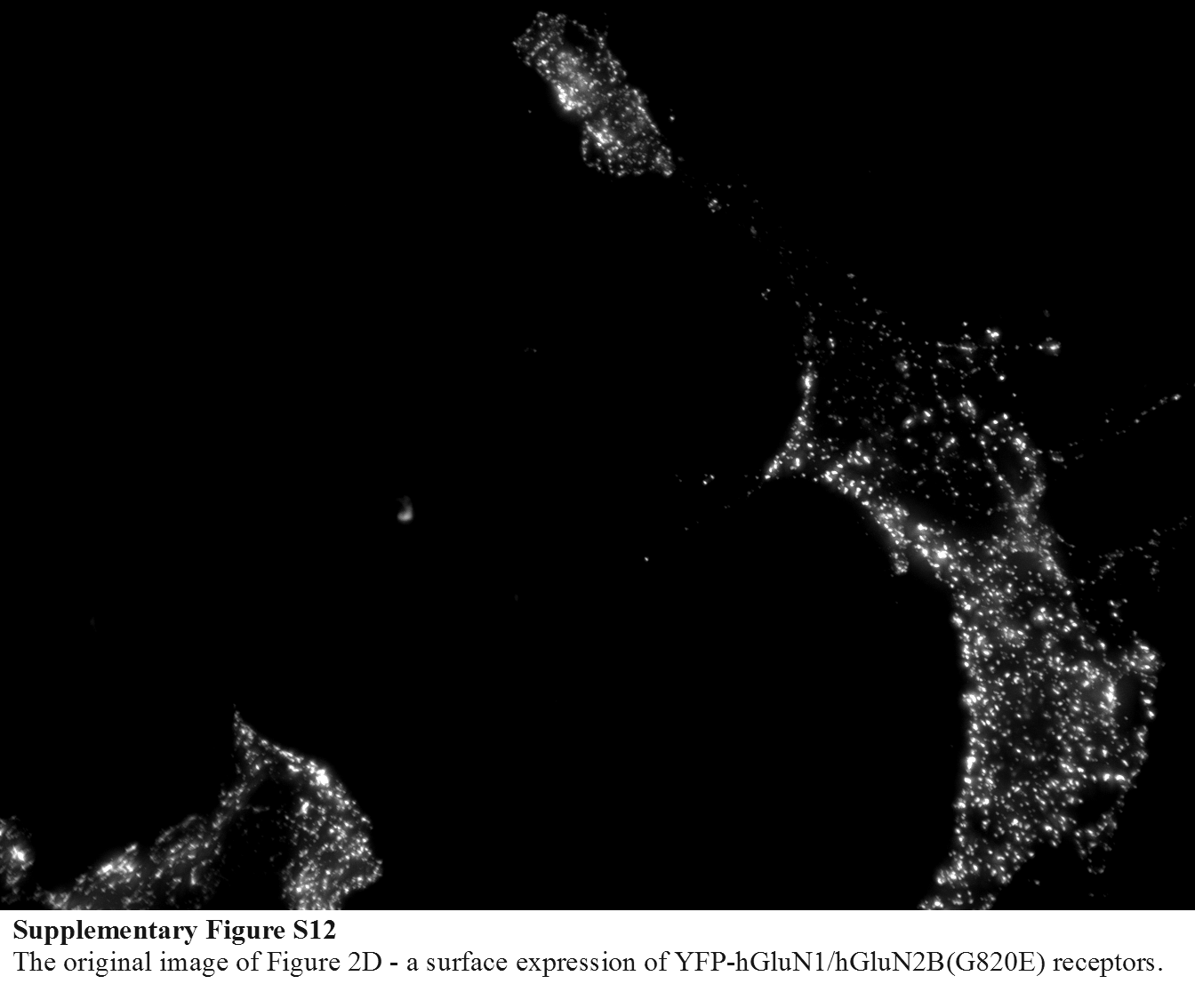

Supplement: Supplementary file 9 [file Image12.tif]
